# Multiuser all-optical quantum network based on metasurfaces

**DOI:** 10.1126/sciadv.adu8455

**Published:** 2025-10-10

**Authors:** Shengshuai Liu, Lin Li, Yujie Wang, Minghao Ning, Yanbo Lou, Yingxuan Chen, Rui Zhang, Jiabin Wang, Qinmiao Chen, Quan Yuan, Shuming Wang, Shumin Xiao, Din Ping Tsai, Ya Cheng, Shining Zhu, Jietai Jing

**Affiliations:** ^1^ State Key Laboratory of Precision Spectroscopy, Joint Institute of Advanced Science and Technology, School of Physics and Electronic Science, East China Normal University. Shanghai 200062, China.; ^2^ Institute of Laser Manufacturing, Henan Academy of Sciences, Zhengzhou 450046, China.; ^3^ Collaborative Innovation Center of Extreme Optics, Shanxi University, Taiyuan 030006, China.; ^4^ Ministry of Industry and Information Technology Key Lab of Micro-Nano Optoelectronic Information System, Guangdong Provincial Key Laboratory of Semiconductor Optoelectronic Materials and Intelligent Photonic Systems, Harbin Institute of Technology, Shenzhen, China.; ^5^ National Laboratory of Solid State Microstructures, School of Physics, Nanjing University, Nanjing 210093, China.; ^6^ Pengcheng Laboratory, Shenzhen, Guangdong 518055, China.; ^7^ Department of Electrical Engineering, City University of Hong Kong, Kowloon, Hong Kong SAR, China.; ^8^ CAS Center for Excellence in Ultra-intense Laser Science, Shanghai 201800, China.

## Abstract

A crucial aspect of quantum information is the establishment of multiuser quantum networks, ensuring secure transmission of information among separated users. However, establishing a large-scale network remains a substantial challenge, requiring massive and compact Einstein-Podolsky-Rosen (EPR) entangled states. Here, we experimentally generate a 5 by 5 continuous variable (CV) EPR entanglement array using a metalens array. Moreover, on the basis of such a compact EPR entanglement array, we establish a five-user all-optical quantum state sharing (AOQSS) network with fidelity beating the corresponding classical limit, which is currently the largest AOQSS network in the CV regime. These results provide a promising platform for the generation of massive and compact EPR entangled states and the construction of large-scale all-optical multiuser quantum networks. Our compact approach for generating CV EPR entanglement based on metasurface opens up avenues for advanced quantum networks.

## Teaser

We experimentally demonstrate a multiuser all-optical quantum network on the basis of metasurfaces.

## INTRODUCTION

Quantum information (*1*) has garnered global attention for its potential to substantially enhance information processing capacity and security through the exploitation of quantum effects. In quantum information, there are two important portions, i.e., discrete variable (DV) (*2*) and continuous variable (CV) (*3*) quantum systems. The DV quantum system uses physical quantity with a discrete spectrum to describe the quantum state, offering the advantage of resistance against losses (*2*). Differently, the CV quantum system uses physical quantity with a continuous spectrum to describe the quantum state and has the advantage of deterministic implementation (*3*). In other words, the CV quantum system and the DV quantum system are different in physical nature and thus have different advantages. The advancement of quantum information research has led to the development of various protocols in DV and CV quantum systems, including quantum teleportation (*4*–*12*), quantum key distribution (*13*, *14*), quantum cloning (*15*–*19*), etc. The scale of quantum information protocols is primarily determined by the scale of quantum resources, notably the number of coexisting Einstein-Podolsky-Rosen (EPR) (*20*) entangled states in a single quantum system. Therefore, for the establishment of a large-scale quantum network, it has become an urgent issue to generate massive and compact EPR entangled states.

In contrast to conventional bulky optical components, metasurfaces offer compact and planar alternatives for optical systems and devices (*21*–*23*), reshaping the photonics landscape in both classical (*24*–*26*) and nonclassical domains (*27*–*34*). Particularly in the DV regime, metasurfaces have successfully established compact high-dimensional (*31*) and complex quantum states (*34*). However, practical applications of quantum entanglement based on metasurfaces have not been realized in the previous work (*31*). Moreover, in the CV regime, EPR entanglement is primarily generated by parametric amplifiers with relatively complex structures (*7*, *35*). Under this condition, it is extremely complex and challenging to generate multiple EPR entanglements using multiple parametric amplifiers, limiting their realistic applications in quantum networks. In this sense, there is a pressing need to explore a compact and sophisticated method for generating massive CV EPR entanglement and to facilitate the construction of large-scale quantum networks.

**Fig. 1.**
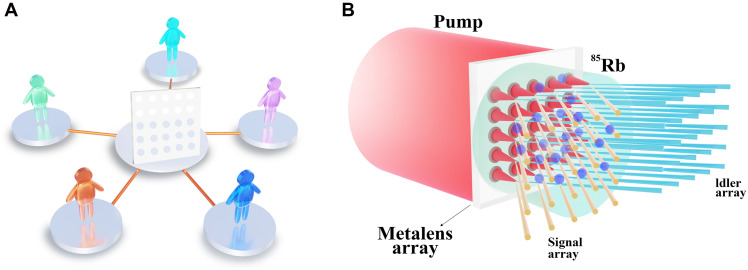
Schematic of the experiment. (**A**) The schematic of five-user AOQSS network. (**B**) The setup for generating EPR entanglement array. ^85^Rb, ^85^Rb vapor cell.

Here, we present the experimental demonstration of an EPR entanglement array based on a metalens array and construct a five-user all-optical quantum state sharing (AOQSS) network (*36*) using the generated EPR state (Fig. 1A). Through a 5 by 5 metalens array and an ^85^Rb vapor cell, we achieve a 5 by 5 CV EPR entanglement array via a double-Λ configuration four-wave mixing (FWM) process (*35*, *37*, *38*). Notably, the implementation of such a compact array poses challenges with conventional optical components (see metalens design details in the Supplementary Materials). The CV EPR entanglements facilitated by the metalens array are harnessed to construct a five-user AOQSS network (*36*) with fidelity beating the corresponding classical limit, which is currently the largest AOQSS network in the CV regime.

## RESULTS

**Fig. 2.**
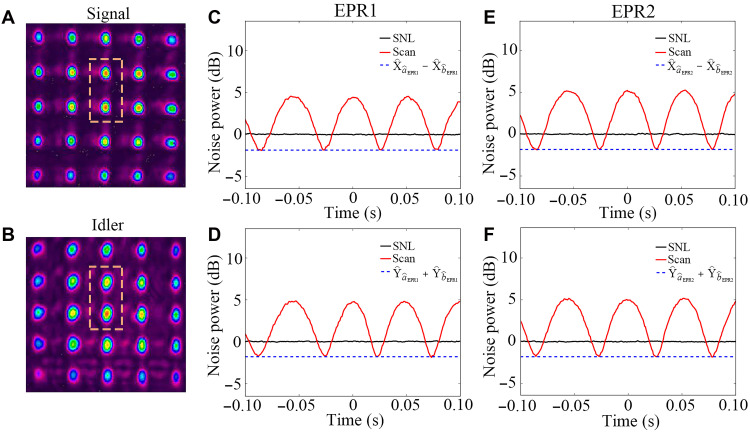
The typical noise power results for EPR array. (**A**) and (**B**) are the images of signal array and idler array generated from FWM processes. (**C**) and (**D**) show the amplitude quadrature difference and phase quadrature sum of EPR1, respectively. The variance of amplitude quadrature difference (phase quadrature sum) is the blue dashed line. The noise power of the photocurrent output from balanced homodyne detection (BHD) by scanning the relative phase between ${\hat{a}}_{\text{EPR}1}$ and ${\hat{b}}_{\text{EPR}1}$ is red curve. (**E**) and (**F**) show the amplitude quadrature difference and phase quadrature sum of EPR2, respectively.

The experimental schematic for generating the EPR entanglement (*20*) array is illustrated in Fig. 1B. A pump laser beam with a frequency of ~1 GHz blue detuned from the D1 line (5*S*_1/2_, *F* = 2 → 5*P*_1/2_; 795 nm) of ^85^Rb is directed onto the 5 by 5 metalens array. Subsequently, the modulated pump beam undergoes division and focusing to produce 5 by 5 spots (see details in the Supplementary Materials) in the center of a 12-mm-long hot ^85^Rb vapor cell. The temperature of ^85^Rb vapor cell is about 118°C. Each of these pump spots has the capability to initiate a spontaneous FWM process, wherein two pump photons convert into one signal photon (redshifted by about 3.04 GHz from the pump beam) and one idler photon (blueshifted by about 3.04 GHz from the pump beam). The resulting signal beams and idler beams exhibit EPR entanglement, thus generating a 5 by 5 CV EPR entanglement array (*35*, *37*, *38*). Here, each metalens is designed with a uniform focal length of about 3 cm at the operational wavelength of 795 nm, covering an area of 300 μm. The metalens array is constructed from TiO_2_ nanocylinders with a uniform height of 1200 nm and variable diameters, fabricated using modified complementary metal-oxide semiconductor (CMOS)-compatible processes including e-beam lithography (EBL) and reactive ion etching (RIE). The focusing efficiency of the metalenses is 47.3 ± 3.3%, and the focusing length is 2.99 ± 0.03 cm. Using the metalens array, a signal array and an idler array are obtained, as captured by the charge-coupled device (CCD) camera, and are presented in Fig. 2 (A and B), respectively.

To validate the EPR entanglement within the array, we use the criterion of inseparability (*39*, *40*), which can be given as $I_{{\hat{a}}_{j},{\hat{b}}_{j}}=\text{Var}\left({\hat{X}}_{{\hat{a}}_{j}}-{\hat{X}}_{{\hat{b}}_{j}}\right)+\text{Var}\left({\hat{Y}}_{{\hat{a}}_{j}}+{\hat{Y}}_{{\hat{b}}_{j}}\right)$, where ${\hat{X}}_{{\hat{a}}_{j}}=\left({\hat{a}}_{j}^{†}+{\hat{a}}_{j}\right)$ $\left[{\hat{X}}_{{\hat{b}}_{j}}=({\hat{b}}_{j}^{†}+{\hat{b}}_{j})\right]$ and ${\hat{Y}}_{{\hat{a}}_{j}}=i\left({\hat{a}}_{j}^{†}-{\hat{a}}_{j}\right)\left[{\hat{Y}}_{{\hat{b}}_{j}}=i\left({\hat{b}}_{j}^{†}-{\hat{b}}_{j}\right)\right]$ are the amplitude and phase quadratures of the corresponding fields. When $I_{{\hat{a}}_{j},{\hat{b}}_{j}}<4$ (*39*, *40*), the two optical fields ${\hat{a}}_{j}$ and ${\hat{b}}_{j}$ are entangled. The balanced homodyne detections (BHDs) are used to measure amplitude quadrature variance and phase quadrature variance of ${\hat{a}}_{j}$ and ${\hat{b}}_{j}$. The corresponding quadrature variance results for two pairs of EPR entanglements enclosed by the orange dashed lines in Fig. 2 (A and B) (EPR1 and EPR2) are shown in Fig. 2 (C to F). The variances of amplitude quadrature difference ${\hat{X}}_{{\hat{a}}_{\text{EPR}1}}-{\hat{X}}_{{\hat{b}}_{\text{EPR}1}}$ and phase quadrature sum ${\hat{Y}}_{{\hat{a}}_{\text{EPR}1}}+{\hat{Y}}_{{\hat{b}}_{\text{EPR}1}}$ of the EPR1 (blue dashed lines) are 1.89 ± 0.18 and 1.79 ± 0.20 dB below the corresponding shot-noise limit (SNL; black lines) as shown in Fig. 2 (C and D), respectively. SNLs used for normalization are the variances of corresponding vacuum fields, which are measured by blocking the measured beams of two BHDs. The corresponding results for EPR2 are shown in Fig. 2 (E and F), which give the values of 1.82 ± 0.18 and 1.80 ± 0.19 dB below the corresponding SNLs for Var$\left({\hat{X}}_{{\hat{a}}_{\text{EPR}2}}-{\hat{X}}_{{\hat{b}}_{\text{EPR}2}}\right)$ and Var$\left({\hat{Y}}_{{\hat{a}}_{\text{EPR}2}}+{\hat{Y}}_{{\hat{b}}_{\text{EPR}2}}\right)$, respectively. In addition, in the CV regime, the covariance matrix (CM) σ is needed to provide a more complete picture of the entanglement (*40*–*42*). For two beams $\hat{a}$ and $\hat{b}$, the CM is expressed as $\sigma =\left\langle {\hat{\delta }}^{T}\hat{\delta }\right\rangle$, where $\hat{\delta }=\left({\hat{X}}_{\hat{a}},{\hat{Y}}_{\hat{a}},{\hat{X}}_{\hat{b}},{\hat{Y}}_{\hat{b}}\right)$. The logarithmic negativity $E_{𝒩}$ can be obtained through the symplectic values of the partially transposed CM $\tilde{\sigma }$ (*41*). $\tilde{\sigma }$ is transformed from σ by the partially transposed operation, which turns ${\hat{Y}}_{\hat{b}}$ into $-{\hat{Y}}_{\hat{b}}$ for the desired subset of modes. The logarithmic negativity can be expressed as $E_{𝒩}=-\sum_{i:{\tilde{\nu }}_{i}<1}\text{ln}{\tilde{\nu }}_{i}$ (*41*), where ${\tilde{\nu }}_{i}$ denotes the symplectic eigenvalue of $\tilde{\sigma }$. If $E_{𝒩}=0$, then it represents a separable state; otherwise, an entangled state. Moreover, the positivity under partial transposition (PPT) criterion (*40*, *42*) is a sufficient and necessary criterion for two-mode Gaussian states. If the smallest symplectic eigenvalue ${\tilde{\nu }}_{\text{min}}$ of partially transposed CM$\;\tilde{\sigma }\;$ is smaller than 1, then there is entanglement between two beams $\hat{a}$ and $\hat{b}$; otherwise, they are separable. In our experiment, the CM can be obtained from the BHDs (*43*). On the basis of the measured CM of EPR1, four eigenvalues of $\tilde{\sigma }$ are 2.89 ± 0.01, 2.89 ± 0.01, 0.65 ± 0.01, and 0.65 ± 0.01, respectively. The corresponding smallest eigenvalue ${\tilde{\nu }}_{\text{EPR}1,\text{min}}$ is 0.65 ± 0.01 < 1, which proves the existence of entanglement between two modes of EPR1 according to PPT criterion. On the basis of these eigenvalues, the logarithmic negativity $E_{{𝒩}_{\text{EPR}1}}$ = 0.86 ± 0.02 > 0, which also demonstrates the entanglement between two modes. Similarly, the partially transposed CM $\tilde{\sigma }$ of EPR2 is experimentally obtained, whose four eigenvalues are 3.25 ± 0.01, 3.25 ± 0.01, 0.65 ± 0.01, and 0.65 ± 0.01, respectively. The ${\tilde{\nu }}_{\text{EPR}2,\text{min}}$ is 0.65 ± 0.01 < 1, and the logarithmic negativity $E_{{𝒩}_{\text{EPR}2}}$ = 0.86 ± 0.02 > 0, which demonstrates the existence of the entanglement. The purity μ of the quantum state can be defined as $\mu =\frac{1}{\sqrt{\text{det}\sigma }}$ (*41*), which is related to the CM and $\text{det}\sigma =\pi_{i}\nu_{i}^{2}$, where $\nu_{i}$ is the symplectic eigenvalue of σ. The purity μ<1 represents that the quantum state is a mixed state, and a for pure state, μ=1. On the basis of the measured CMs of EPR1 and EPR2, the purities of EPR1 and EPR2 are $\mu_{\text{EPR}1}$ = 28.3 ± 0.6% and $\mu_{\text{EPR}2}$ = 22.4 ± 0.5%, respectively. Thus, EPR1 and EPR2 in our experiment are both mixed states. These results indicate that we generate a 5 by 5 CV EPR entanglement array using only one nonlinear medium (see details in the Supplementary Materials). Moreover, this approach has the potential for easy extension to larger arrays. In contrast, the conventional EPR generation method would require multiple nonlinear media and numerous bulky components to produce such an EPR entanglement array. In addition, this method presents substantial challenges for other traditional compact optical devices (see details in the Supplementary Materials).

**Fig. 3.**
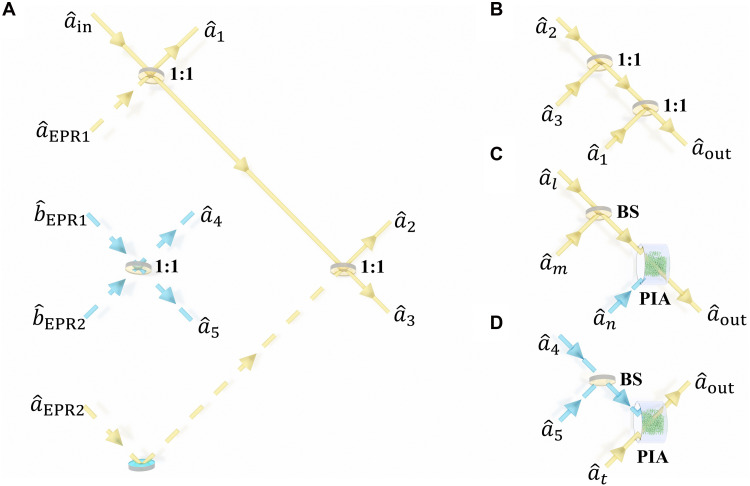
Setup of five-user AOQSS network. (**A**) The dealer protocol. ${\hat{a}}_{\text{in}}$: The annihilation operator associated with the secret state. ${\hat{a}}_{\text{EPR}1}$, ${\hat{b}}_{\text{EPR}1}$, ${\hat{a}}_{\text{EPR}2}$, and ${\hat{b}}_{\text{EPR}2}$: The annihilation operators associated with two pairs of EPR entanglement in entanglement array. 1:1: 50:50 BS. ${\hat{a}}_{1}$, ${\hat{a}}_{2}$, ${\hat{a}}_{3}$, ${\hat{a}}_{4}$, and ${\hat{a}}_{5}$: The annihilation operators associated with the five shares. (**B**) The {1, 2, 3} reconstruction structure. ${\hat{a}}_{\text{out}}$: The annihilation operator associated with the output recovered state. (**C**) The reconstruction structure with two users in ${\hat{a}}_{1}$, ${\hat{a}}_{2}$, or ${\hat{a}}_{3}$ and one user in ${\hat{a}}_{4}$ or ${\hat{a}}_{5}$. (**D**) The reconstruction structure with ${\hat{a}}_{4}$ or ${\hat{a}}_{5}$, and one user in ${\hat{a}}_{1}$, ${\hat{a}}_{2}$, or ${\hat{a}}_{3}$.

To demonstrate that the metalens-array–based entanglement has the applicability in increasing the scale of the quantum network, a five-user AOQSS network (*36*) is constructed. In such a five-user AOQSS network [threshold deterministic AOQSS (*3*, *5*)], the secret state can be reconstructed by any three users. The schematic of the five-user AOQSS network is shown in Fig. 3. The secret state is denoted as ${\hat{a}}_{\text{in}}$ in Fig. 3A, which is red shifted by 3.04 GHz from the pump beam and has a power of about 0.3 μW. This state is obtained by passing a laser beam through an acousto-optic modulator. Then, ${\hat{a}}_{\text{in}}$ and ${\hat{a}}_{\text{EPR}1}$ are combined by a 50:50 beam splitter (BS). One output of this BS denotes ${\hat{a}}_{1}$. The other output of this BS and ${\hat{a}}_{\text{EPR}2}$ are combined by another 50:50 BS, obtaining ${\hat{a}}_{2}$ and ${\hat{a}}_{3}$. ${\hat{a}}_{4}$ and ${\hat{a}}_{5}$ are obtained by combining ${\hat{b}}_{\text{EPR}1}$ and ${\hat{b}}_{\text{EPR}2}$ with 50:50 BS. Then, ${\hat{a}}_{1}$ to ${\hat{a}}_{5}$ are sent to five users, respectively. In our experiment, the EPR generation process is based on the FWM process in which some unwanted nonlinear processes can bring additional Gaussian noise into the amplitude quadrature and phase quadrature of generated EPR entanglement. In this way, when the secret state is combined with generated EPR entanglements, the additional Gaussian noise is introduced naturally into our AOQSS (*44*). Moreover, as mentioned in (*36*, *44*), additional Gaussian noise is encoded onto each share. If the EPR entanglement degree used in AOQSS approaches infinity, then the variance of each share is also infinity. In this case, a single player (user) cannot get any information about the secret state, and additional Gaussian noise is irrelevant to the protocol security. However, in the practical experiment, the noises of used EPR entangled beams are finite. The information about the secret state obtained by a single user can be covered by such additional Gaussian noise to some extent, enhancing the security of AOQSS. Therefore, in the practical experiment, higher Gaussian noise levels correspond to greater protocol security. The reconstruction structures of (*3*, *5*) threshold deterministic AOQSS are shown in Fig. 3 (B to D). In the {1, 2, 3} reconstruction structure shown in Fig. 3B, the secret state can be reconstructed by combining ${\hat{a}}_{1}$, ${\hat{a}}_{2}$, and ${\hat{a}}_{3}$ by two 50:50 BSs. For other reconstruction structures, the secret state can be reconstructed in two steps. First, two shares are combined by a BS with a special transmissivity. Subsequently, the output of the BS and the remaining share are sent to a phase-insensitive amplifier (PIA) with a special intensity gain based on the FWM process (*12*, *38*, *45*). In all reconstruction configurations of our scheme, the power of the reconstructed state ${\hat{a}}_{\text{out}}$ is about 0.3 μW, which equals the input state (*19*). To check the performance of AOQSS, fidelity F is used, which is defined as $F=\left\langle \Psi_{\text{in}}\;|\rho_{\text{out}}\;|\Psi_{\text{in}}\right\rangle$ (*46*). The AOQSS is successful if the average fidelity surpasses the corresponding classical limit of ^3^/_5_ (*36*, *47*). A detailed derivation and experimental scheme for the (*3*, *5*) threshold deterministic AOQSS protocol are presented in the Supplementary Materials.

**Fig. 4.**
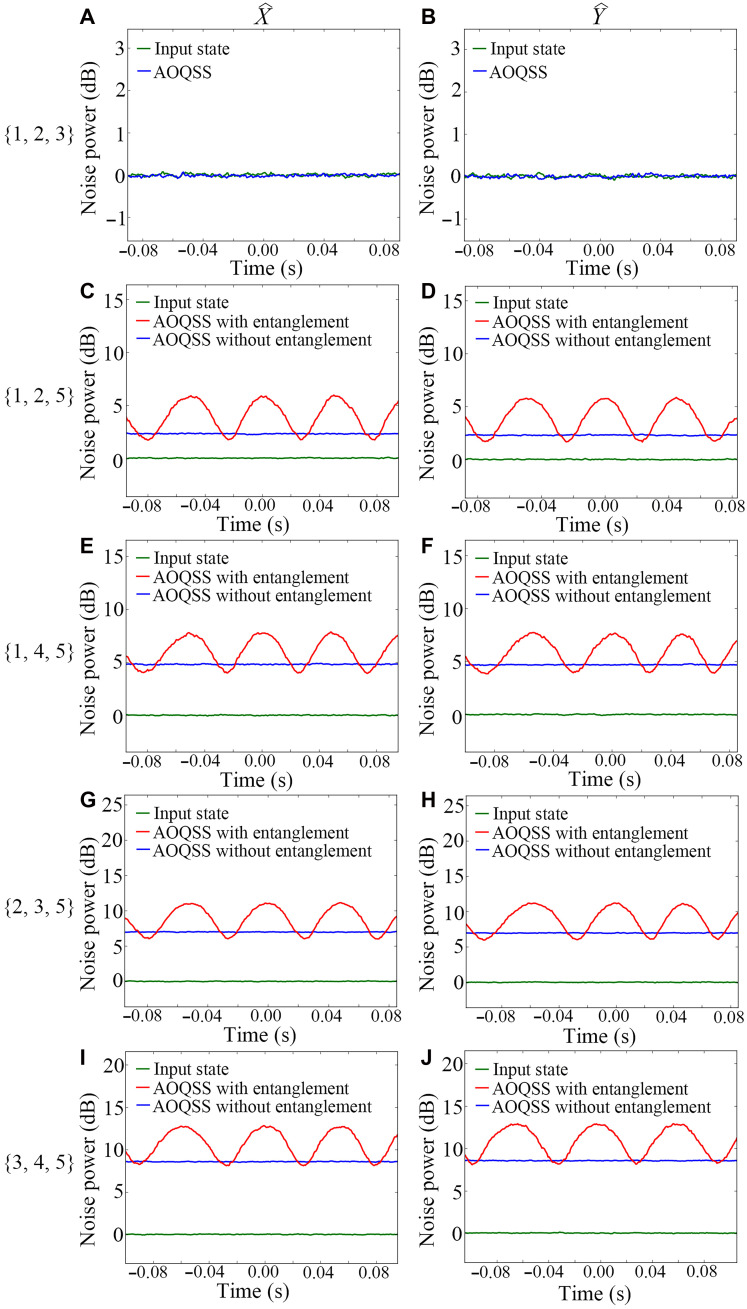
The noise power results for reconstruction structures. (**A** and **B**) The amplitude (phase) quadrature variance for the input secret state and output recovered state of {1, 2, 3} reconstruction structure. (**C** and **D**) The amplitude (phase) quadrature variance of {1, 2, 5} reconstruction structure. (**E** and **F**) The amplitude (phase) quadrature variance of {1, 4, 5} reconstruction structure. (**G** and **H**) The amplitude (phase) quadrature variance of {2, 3, 5} reconstruction structure. (**I** and **J**) The amplitude (phase) quadrature variance of {3, 4, 5} reconstruction structure. The green traces are the quadrature variances of the input secret state. The blue and red traces are the quadrature variances of the output recovered state without and with entanglement, respectively.

The reconstructed state ${\hat{a}}_{\text{out}}$ is measured by a BHD to evaluate its quality. Figure 4 shows the typical noise power results for various reconstruction structures. The green and blue traces in Fig. 4 (A and B) represent the amplitude and phase quadrature variances for the input secret state ${\hat{a}}_{\text{in}}$ and the reconstructed output state ${\hat{a}}_{\text{out}}$, respectively. We can see that the green traces are almost equal to the blue traces, which gives the fidelity of the {1, 2, 3} reconstruction structure, i.e., about 1. In other words, we almost retrieve the secret state. For the {1, 2, 5} reconstruction structure shown in Fig. 4 (C and D), the green traces are the quadrature variances of the input secret state ${\hat{a}}_{\text{in}}$, while the blue and red traces give the quadrature variances of the output recovered state ${\hat{a}}_{\text{out}}$ without and with entanglement, respectively. It is evident that the introduction of EPR entanglement decreases the noise of ${\hat{a}}_{\text{out}}$. The relative phase between ${\hat{a}}_{\text{EPR}1}$ and ${\hat{b}}_{\text{EPR}1}$ (${\hat{a}}_{\text{EPR}2}$ and ${\hat{b}}_{\text{EPR}2}$) is scanned by a piezo-electric transducer. The minimum value of red traces means that the relative phase between ${\hat{a}}_{\text{EPR}1}$ and ${\hat{b}}_{\text{EPR}1}$ (${\hat{a}}_{\text{EPR}2}$ and ${\hat{b}}_{\text{EPR}2}$) corresponds to ${\hat{X}}_{{\hat{a}}_{\text{EPR}1}}-{\hat{X}}_{{\hat{b}}_{\text{EPR}1}}$ and ${\hat{Y}}_{{\hat{a}}_{\text{EPR}1}}+{\hat{Y}}_{{\hat{b}}_{\text{EPR}1}}$ $\left(\;{\hat{X}}_{{\hat{a}}_{\text{EPR}2}}-{\hat{X}}_{{\hat{b}}_{\text{EPR}2}}\right.$ and $\left.{\hat{Y}}_{{\hat{a}}_{\text{EPR}2}}+{\hat{Y}}_{{\hat{b}}_{\text{EPR}2}}\;\right)$. Therefore, we can treat the minima of red traces as the variances of the reconstructed output state. On the basis of the green and red traces in Fig. 4 (C and D), the fidelity of AOQSS with {1, 2, 5} reconstruction structure is 0.81 ± 0.01. {1, 2, 4}, {1, 3, 4}, and {1, 3, 5} are equivalent to {1, 2, 5} reconstruction structure as shown in the Supplementary Materials, and the fidelities of the reconstructed output state in these reconstruction structures are 0.80 ± 0.01, 0.81 ± 0.01, and 0.81 ± 0.01, respectively. Meanwhile, for the {1, 4, 5} reconstruction structure shown in Fig. 4 (E and F), the fidelity of the reconstructed output state is 0.58 ± 0.01. For the {2, 3, 5} ({3, 4, 5}) reconstruction structure shown in Fig. 4 (G and H) (I and J), the measured fidelity is 0.40 ± 0.02 (0.27 ± 0.01), while {2, 3, 4} ({2, 4, 5}) exhibits an equivalent fidelity of 0.40 ± 0.01 (0.27 ± 0.01) to {2, 3, 5} ({3, 4, 5}). On the basis of all these results, the average fidelity for (*3*, *5*) threshold AOQSS is 0.62 ± 0.01, beating the corresponding classical limit of ^3^/_5_. This indicates the successful experimental implementation of (*3*, *5*) threshold AOQSS based on two pairs of EPR entanglement in the entanglement array. The fidelity of our AOQSS protocol is mainly limited by the noise introduced by the PIAs based on FWM processes for generating EPR entanglement and reconstructing the secret state. The Glan-Thompson polarizer is used to eliminate the residual pump beam of the FWM process. The Glan-Thompson polarizer with an imperfect extinction ratio will introduce the noise of the scattering pump beam into the AOQSS protocol. In the future, we can replace the currently used Glan-Thompson polarizer with the one with a better extinction ratio. This can suppress the amount of pump scattering to some extent, enhancing the fidelity. Moreover, the squeezing level of the generated EPR states with the metalens-based approach is somewhat reduced compared to the results without using metalens. This is mainly because the angle between the signal and pump beams in the metalens-based approach is set to 10 mrad instead of the optimal 7 mrad (*48*). This adjustment was made to balance the metasurface area and the processing time required for our EBL fabrication. Advanced fabrication technologies, such as high-energy EBL or deep ultraviolet lithography (*49*, *50*), are expected to greatly improve fabrication speed and stability for large-area metasurfaces. Under these improved conditions, the fidelity can be substantially improved by selecting the optimal angles with higher squeezing levels. In addition, the metasurface array can be substantially expanded to enable larger-scale EPR entangled states, which hold great promise for advanced quantum applications.

## DISCUSSION

As mentioned in (*47*, *51*), to further scale up the AOQSS network, the dealer requires an increasing number of EPR entangled sources to conceal the secret coherent state. Using the entanglement resources within the 5 by 5 EPR entanglement array to their fullest potential enables the experimental realization of a large-scale AOQSS network. With the increase in AOQSS network scale, the complexity of the optical setup will increase. Notably, recent progresses in metasurfaces provide effective solutions for compact integration and miniaturization of various optical functionalities, or even combinations of multiple functionalities (*52*–*54*), that were traditionally achieved using bulky optical components. Consequently, beyond the realization of large-scale EPR entanglement in this study, metasurfaces offer the potential of greatly simplifying and optimizing the construction of quantum networks, which is promising for large-scale quantum network applications. Moreover, the quantum communication network can also be experimentally constructed by several EPR entanglement states and multiplexing technology (*55*). Recently, enabled by the all-optical structure and four degrees of freedom (DOFs) multiplexing technology, deterministic all-optical quantum teleportation of four DOFs has been achieved (*56*). In forthcoming research, by combining multi-DOF multiplexing technology with compact CV EPR entanglement technology, the scale of the quantum communication network can be substantially enhanced. These advancements show the substantial potential of the compact approach for EPR entanglement generation based on a metalens array, offering substantial promise for scaling up quantum networks.

In summary, we have successfully generated a 5 by 5 CV EPR entanglement array using a metalens array. Our experimental results showcase the effectiveness of constructing a five-user AOQSS network with these EPR entanglements, which is the largest AOQSS network in the CV regime to date. Across various reconstruction structures, the average fidelity is 0.62 ± 0.01, surpassing the corresponding classical limit. This accomplishment not only validates the feasibility of metalens-array–based CV EPR entanglements but also provides a promising foundation for the realization of large-scale, all-optical multiuser quantum networks. Our compact method of generating CV EPR entanglement based on metasurface paves the way for the development of extensive and advanced quantum communication networks.

## MATERIALS AND METHODS

### Sample fabrication

The sample was manufactured using a CMOS process, which has some modifications in special steps. The metasurface pattern was exposed by EBL on a quartz substrate with a 1200-nm-thick titanium dioxide (TiO_2_) film. The quartz substrate was cleaned by ultrasonic wave in acetone, methanol, and isopropanol in sequence. After cleaning, TiO_2_ film with a thickness of 1200 nm was deposited onto the substrate with electron-beam evaporation. Subsequently, 200-nm-thick polymethyl methacrylate (PMMA) A2 (MicroChem) electron-beam resist was spin coated onto the TiO_2_ surface. The resist was exposed through EBL with a beam current of 20 pA to define the inversed structure. The patterns are revealed after the development process in methyl isobutyl ketone/isopropyl alcohol for 50 s. A 30-nm Cr layer was highly directionally deposited onto the inversed pattern via electron-beam evaporation. Then, the PMMA resist was removed in photoresist remover solution, and the metasurface pattern was transferred into the Cr hard mask. Next, the TiO_2_ film was etched by RIE with SF_6_, CHF_3_, Ar, and O_2_ gas. The final metasurface sample was obtained after removing the Cr hard mask using chrome etchant. The detailed process is shown in fig. S5. In addition, a comprehensive discussion of the fabrication tolerance is also presented in the “Fabrication tolerance” section in the Supplementary Materials.

### Characterization of the metalens array

After fabrication of the metalens array, we performed the characterization of the sample using a home-built microscopy system. In the system, a pump laser with a wavelength of 795 nm is directed at the metalens array sample, and the modulated light is collected by an objective lens (10×, numerical aperture = 0.26). The light intensity information is subsequently recorded by a CCD camera. The metasurface sample is mounted on a high-precision three-dimensional displacement platform. The truncated images of planes at various distances from the sample are recorded by the CCD camera. Last, these recorded images enable the reconstruction of the intensity profile of the focus in the *x*-*z* plane. The focusing efficiency, the focusing profile, the focal length, and the full width at half maximum of the focuses produced by the metalens array are measured with this setup. Detailed descriptions of the characterization system and the measured data can be found in the “Characterization of the metalens array” section in the Supplementary Materials.

## Supplementary Materials

### This PDF file includes:

Supplementary Text Figs. S1 to S8 Tables S1 and S2 References
