# Supplementary material for: Multiuser all-optical quantum network based on metasurfaces
Source: Sci Adv. 2025 Oct 10;11(41):eadu8455. doi: 10.1126/sciadv.adu8455 (PMC13155480; doi:10.1126/sciadv.adu8455)
Supplement: Supplementary file 1 — Supplementary Text Figs. S1 to S8 Tables S1 and S2 References [file sciadv.adu8455_sm.pdf]

Science Advances
AAAS

Supplementary Materials for

Multiuser all-optical quantum network based on metasurfaces

Shengshuai Liu *et al.*

Corresponding author: Lin Li, lli@lps.ecnu.edu.cn; Shumin Xiao, shumin.xiao@hit.edu.cn; Din Ping Tsai, dptsai@cityu.edu.hk; Jietai Jing, jtjing@phy.ecnu.edu.cn

Sci. Adv. **11**, eadu8455 (2025)
DOI: 10.1126/sciadv.adu8455

This PDF file includes:

Supplementary Text
Figs. S1 to S8
Tables S1 and S2
References

Supplementary Text

(3, 5) threshold deterministic all-optical quantum state sharing (AOQSS) protocol

In the (3, 5) threshold AOQSS, the dealer encodes a secret state into five shares and subsequently distributes them to five users. The reconstruction of the secret state can be achieved through the collaborative efforts of any three users. The structure of (3, 5) threshold deterministic AOQSS protocol is shown in Fig. S1. Firstly, the secret coherent state $\hat{a}_{\text{in}}$ is encoded into five shares with the help of two Einstein-Podolsky-Rosen (EPR) entangled sources. The Hamiltonians of these EPR entangled sources generated by four-wave mixing (FWM) processes can be given by

$$\begin{aligned}\hat{H}_1 &= i\hbar r_1 \hat{a}^{\dagger}_{\text{EPR1}} \hat{b}^{\dagger}_{\text{EPR1}} + \text{ H.c.},\\ \hat{H}_2 &= i\hbar r_2 \hat{a}^{\dagger}_{\text{EPR2}} \hat{b}^{\dagger}_{\text{EPR2}} + \text{ H.c..}\end{aligned} \tag{S1}$$

$\hat{a}^{\dagger}_{\text{EPR1}}$ and $\hat{b}^{\dagger}_{\text{EPR1}}$ ($\hat{a}^{\dagger}_{\text{EPR2}}$ and $\hat{b}^{\dagger}_{\text{EPR2}}$) are the creation operators associated with entangled source EPR1 (EPR2). r_1 and r_2 denote the interaction strengths of FWM processes. H.c. is the Hermitian conjugate. The two EPR entangled sources can be given by (*38*)

$$\begin{aligned}\hat{a}_{\text{EPR1}}(\tau_1) &= \sqrt{G_1}\hat{a}_{01} + \sqrt{G_1 - 1}\hat{b}^{\dagger}_{01},\\ \hat{b}^{\dagger}_{\text{EPR1}}(\tau_1) &= \sqrt{G_1 - 1}\hat{a}_{01} + \sqrt{G_1}\hat{b}^{\dagger}_{01},\\ \hat{a}_{\text{EPR2}}(\tau_2) &= \sqrt{G_2}\hat{a}_{02} + \sqrt{G_2 - 1}\hat{b}^{\dagger}_{02},\\ \hat{b}^{\dagger}_{\text{EPR2}}(\tau_2) &= \sqrt{G_2 - 1}\hat{a}_{02} + \sqrt{G_2}\hat{b}^{\dagger}_{02},\end{aligned} \tag{S2}$$

where $\hat{a}_{01}$, $\hat{b}_{01}$, $\hat{a}_{02}$, and $\hat{b}_{02}$ represent vacuum inputs of FWM processes. $G_1 = \cosh^2(r_1\tau_1)$ and $G_2 = \cosh^2(r_2\tau_2)$ are the intensity gains of the FWM processes. τ_1 (τ_2) is the interaction time. As shown in Fig. S1A, by combining $\hat{a}_{\text{in}}$ with two EPR entangled sources through three 50:50 beam splitters (BSs), the five shares can be obtained and expressed as

$$\begin{aligned}\hat{a}_1 &= \frac{1}{\sqrt{2}}(\hat{a}_{\text{in}} + \hat{a}_{\text{EPR1}} + \delta N_1),\\ \hat{a}_2 &= \frac{1}{2}\hat{a}_{\text{in}} - \frac{1}{2}\hat{a}_{\text{EPR1}} - \frac{1}{\sqrt{2}}\hat{a}_{\text{EPR2}} - \frac{1}{2}\delta N_1 - \frac{1}{\sqrt{2}}\delta N_2,\\ \hat{a}_3 &= \frac{1}{2}\hat{a}_{\text{in}} - \frac{1}{2}\hat{a}_{\text{EPR1}} + \frac{1}{\sqrt{2}}\hat{a}_{\text{EPR2}} - \frac{1}{2}\delta N_1 + \frac{1}{\sqrt{2}}\delta N_2,\\ \hat{a}_4 &= \frac{1}{\sqrt{2}}\left(\hat{b}_{\text{EPR2}} - \hat{b}_{\text{EPR1}} + \delta N_2{}^* - \delta N_1{}^*\right),\\ \hat{a}_5 &= \frac{1}{\sqrt{2}}\left(\hat{b}_{\text{EPR2}} + \hat{b}_{\text{EPR1}} + \delta N_2{}^* + \delta N_1{}^*\right),\end{aligned} \tag{S3}$$

where $\hat{a}_1$, $\hat{a}_2$, $\hat{a}_3$, $\hat{a}_4$, and $\hat{a}_5$ are the annihilation operators of five shares. δN_1 and δN_2 denote the additional Gaussian noise. Then, five shares are sent to five users.

For (3, 5) threshold deterministic AOQSS protocol, there are ten reconstruction structures. For {1, 2, 3} reconstruction structure as shown in Fig. S1B, the first 50:50 BS is utilized to combine $\hat{a}_2$ and $\hat{a}_3$. Then, $\hat{a}_1$ and the output of this BS are combined by the second 50:50 BS. In this way, the secret state can be retrieved. The output state $\hat{a}_{\text{out}}$ of the second BS can be given by

$$\hat{a}_{\text{out}} = \frac{1}{\sqrt{2}}\left[\hat{a}_1 + \frac{1}{\sqrt{2}}\left(\hat{a}_2 + \hat{a}_3 e^{i\theta_1}\right)e^{i\theta_2}\right], \tag{S4}$$

where θ_1 is the relative phase between $\hat{a}_2$ and $\hat{a}_3$, and θ_2 is the relative phase between $\hat{a}_1$ and the output of the first BS. When $\theta_1 = 0$ and $\theta_2 = 0$, $\hat{a}_{\text{out}}$ can be given by

$$\hat{a}_{\text{out}} = \frac{1}{\sqrt{2}}\hat{a}_1 + \frac{1}{2}(\hat{a}_2 + \hat{a}_3) = \hat{a}_{\text{in}}. \tag{S5}$$

The amplitude quadrature and phase quadrature of this output state can be expressed as

$$\begin{aligned} \hat{X}_{\text{out}} &= \hat{a}_{\text{out}}^{\dagger} + \hat{a}_{\text{out}} = \hat{X}_{\text{in}}, \\ \hat{Y}_{\text{out}} &= i(\hat{a}_{\text{out}}^{\dagger} - \hat{a}_{\text{out}}) = \hat{Y}_{\text{in}}. \end{aligned} \tag{S6}$$

In our experiment, the relative phase locking is enabled by microcontrol unit (MCU). The fidelity F quantifying the quality of the reconstructed state of deterministic AOQSS protocol is defined as $F = \langle \Psi_{\text{in}} | \rho_{\text{out}} | \Psi_{\text{in}} \rangle$. Here, the input secret state is a coherent state. Considering that all states involved in our scheme are Gaussian, the fidelity can be given by

$$F = 2e^{-(k_x+k_y)/4} / \sqrt{(1+\Delta^2\hat{X}_{\text{out}})(1+\Delta^2\hat{Y}_{\text{out}})}, \tag{S7}$$

where $k_x = \langle \hat{X}_{\text{in}} \rangle^2 (1-g_x)^2/(1+\Delta^2\hat{X}_{\text{out}})$, $k_y = \langle \hat{Y}_{\text{in}} \rangle^2 (1-g_y)^2/(1+\Delta^2\hat{Y}_{\text{out}})$, $g_x = \langle \hat{X}_{\text{out}} \rangle / \langle \hat{X}_{\text{in}} \rangle$, and $g_y = \langle \hat{Y}_{\text{out}} \rangle / \langle \hat{Y}_{\text{in}} \rangle$. For input coherent state $\hat{a}_{\text{in}}$, $\Delta^2\hat{X}_{\text{in}} = \Delta^2\hat{Y}_{\text{in}} = 1$. Based on Eqs. (S6) and (S7), the fidelity of {1, 2, 3} reconstruction structure can be calculated as $F_{\{1,2,3\}}^{\text{clas}} = 1$.

For {1, 4, 5} reconstruction structure as shown in Fig. S1C, $\hat{a}_4$ is combined with $\hat{a}_5$ by a 50:50 BS. Then, with the help of the output of this BS, $\hat{a}_1$ is amplified by a phase-insensitive amplifier (PIA), which can be given by

$$\hat{a}_{\text{out}} = \sqrt{G_3}\hat{a}_1 + \sqrt{\frac{G_3-1}{2}}(\hat{a}_4^{\dagger} + \hat{a}_5^{\dagger}e^{-i\theta_1})e^{i\theta_2}, \tag{S8}$$

where G_3 is the intensity gain of the PIA. θ_1 is the relative phase between $\hat{a}_4$ and $\hat{a}_5$, and θ_2 is the relative phase between $\hat{a}_1$ and the output of the BS. When $\theta_1 = \pi$ and $\theta_2 = 0$, $\hat{a}_{\text{out}}$ can be given by

$$\begin{aligned} \hat{a}_{\text{out}} &= \sqrt{G_3}\hat{a}_1 + \sqrt{\frac{G_3-1}{2}}(\hat{a}_4^{\dagger} - \hat{a}_5^{\dagger}) \\ &= \sqrt{\frac{G_3}{2}}\hat{a}_{\text{in}} + \sqrt{\frac{G_3}{2}}\hat{a}_{\text{EPR1}} - \sqrt{G_3-1}\hat{b}_{\text{EPR1}}^{\dagger} + \left(\sqrt{\frac{G_3}{2}} - \sqrt{G_3-1}\right)\delta N_1. \end{aligned} \tag{S9}$$

Under the condition of $G_3 = 2$, the amplitude quadrature and phase quadrature of $\hat{a}_{\text{out}}$ can be expressed as

$$\begin{aligned} \hat{X}_{\text{out}} &= \hat{X}_{\text{in}} + \hat{X}_{\hat{a}_{\text{EPR1}}} - \hat{X}_{\hat{b}_{\text{EPR1}}}, \\ \hat{Y}_{\text{out}} &= \hat{Y}_{\text{in}} + \hat{Y}_{\hat{a}_{\text{EPR1}}} + \hat{Y}_{\hat{b}_{\text{EPR1}}}. \end{aligned} \tag{S10}$$

The corresponding quadrature variances can be expressed as

$$\Delta^2\hat{X}_{\text{out}} = \Delta^2\hat{Y}_{\text{out}} = 4G_1 - 4\sqrt{G_1(G_1-1)} - 1. \tag{S11}$$

Based on Eq. (S7), the fidelity of {1, 4, 5} reconstruction structure can be calculated as

$$F_{\{1,4,5\}} = 1/\left[2G_1 - 2\sqrt{G_1(G_1-1)}\right]. \tag{S12}$$

When $G_1 = 1$ and $G_3 = 2$, the maximum achievable fidelity of {1, 4, 5} reconstruction structure can be calculated as 1/2. In other words, classical fidelity of {1, 4, 5} reconstruction structure without the help of EPR entangled sources is $F_{\{1,4,5\}}^{\text{clas}} = 1/2$.

For {2, 4, 5} reconstruction structure as shown in Fig. S1D, $\hat{a}_4$ is combined with $\hat{a}_5$ by a BS with a transmissivity of T. Then, with the help of the output of this BS, $\hat{a}_2$ is amplified by a PIA and expressed as

$$\hat{a}_{\text{out}} = \sqrt{G_3}\hat{a}_2 + \sqrt{G_3-1}(\sqrt{T}\hat{a}_5^{\dagger} + \sqrt{1-T}\hat{a}_4^{\dagger}e^{-i\theta_1})e^{i\theta_2}, \tag{S13}$$

where G_3 is the intensity gain of the PIA. θ_1 is the relative phase between $\hat{a}_4$ and $\hat{a}_5$, and θ_2 is the relative phase between $\hat{a}_2$ and the output of the BS. When $\theta_1 = 0$ and $\theta_2 = 0$, $\hat{a}_{\text{out}}$ can be expressed as

$$\begin{aligned}\hat{a}_{\text{out}} &= \sqrt{G_3}\hat{a}_2 + \sqrt{G_3-1}\left(\sqrt{T}\hat{a}_5^\dagger + \sqrt{1-T}\hat{a}_4^\dagger\right)\\
&= \sqrt{\frac{G_3}{4}}\hat{a}_{\text{in}} - \sqrt{\frac{G_3}{4}}\hat{a}_{\text{EPR1}} + \left[\sqrt{\frac{(G_3-1)T}{2}} - \sqrt{\frac{(G_3-1)(1-T)}{2}}\right]\hat{b}_{\text{EPR1}}^\dagger\\
&\quad - \sqrt{\frac{G_3}{2}}\hat{a}_{\text{EPR2}} + \left[\sqrt{\frac{(G_3-1)T}{2}} + \sqrt{\frac{(G_3-1)(1-T)}{2}}\right]\hat{b}_{\text{EPR2}}^\dagger\\
&\quad + \left[-\sqrt{\frac{G_3}{4}} + \sqrt{\frac{(G_3-1)T}{2}} - \sqrt{\frac{(G_3-1)(1-T)}{2}}\right]\delta N_1\\
&\quad + \left[-\sqrt{\frac{G_3}{2}} + \sqrt{\frac{(G_3-1)T}{2}} + \sqrt{\frac{(G_3-1)(1-T)}{2}}\right]\delta N_2.\end{aligned} \tag{S14}$$

Under the condition of $G_3 = 4$ and $T = \sqrt{2}/3 + 1/2$, the amplitude quadrature and phase quadrature of $\hat{a}_{\text{out}}$ can be expressed as

$$\begin{aligned}\hat{X}_{\text{out}} &= \hat{X}_{\text{in}} + \left(\hat{X}_{\hat{b}_{\text{EPR1}}} - \hat{X}_{\hat{a}_{\text{EPR1}}}\right) + g_{1x}\left(\hat{X}_{\hat{b}_{\text{EPR2}}} - \hat{X}_{\hat{a}_{\text{EPR2}}}\right),\\
\hat{Y}_{\text{out}} &= \hat{Y}_{\text{in}} - \left(\hat{Y}_{\hat{a}_{\text{EPR1}}} + \hat{Y}_{\hat{b}_{\text{EPR1}}}\right) - g_{1y}\left(\hat{Y}_{\hat{a}_{\text{EPR2}}} + \hat{Y}_{\hat{b}_{\text{EPR2}}}\right),\end{aligned} \tag{S15}$$

where $g_{1y} = g_{1x} = \sqrt{2}$. The corresponding quadrature variances can be given by

$$\Delta^2\hat{X}_{\text{out}} = \Delta^2\hat{Y}_{\text{out}} = 4G_1 - 4\sqrt{G_1(G_1-1)} - 1 + 2\left[4G_2 - 4\sqrt{G_2(G_2-1)} - 2\right]. \tag{S16}$$

The fidelity of this {2, 4, 5} reconstruction structure can be calculated as

$$F_{\{2,4,5\}} = \frac{1}{2G_1 - 2\sqrt{G_1(G_1-1)} + 2\left[2G_2 - 2\sqrt{G_2(G_2-1)} - 1\right]}. \tag{S17}$$

When $G_1 = 1$, $G_2 = 1$, $G_3 = 4$ and $T = \sqrt{2}/3 + 1/2$, the classical fidelity of {2, 4, 5} reconstruction structure can be calculated as $F_{\{2,4,5\}}^{\text{clas}} = 1/4$. Since the {3, 4, 5} reconstruction structure is equivalent to {2, 4, 5} reconstruction structure, the fidelities of them are equal.

For {1, 2, 4} reconstruction structure as shown in Fig. S1E, $\hat{a}_1$ and $\hat{a}_2$ are combined by a BS with a transmissivity of T. Then, with the help of $\hat{a}_4$, the output of this BS is amplified by a PIA and expressed as

$$\hat{a}_{\text{out}} = \sqrt{G_3}\left(\sqrt{T}\hat{a}_1 + \sqrt{1-T}\hat{a}_2 e^{i\theta_1}\right)e^{i\theta_2} + \sqrt{G_3-1}\hat{a}_4^\dagger, \tag{S18}$$

where G_3 is the intensity gain of the PIA. θ_1 is the relative phase between $\hat{a}_1$ and $\hat{a}_2$, and θ_2 is the relative phase between $\hat{a}_4$ and the output of the BS. When $\theta_1 = 0$ and $\theta_2 = 0$, $\hat{a}_{\text{out}}$ can be given by

$$
\begin{aligned}
\hat{a}_{\text{out}} &= \sqrt{G_3}\left(\sqrt{T}\hat{a}_1 + \sqrt{1-T}\hat{a}_2\right) + \sqrt{G_3 - 1}\hat{a}_4^\dagger \\
&= \left[\sqrt{\frac{G_3 T}{2}} + \sqrt{\frac{G_3(1-T)}{4}}\right]\hat{a}_{\text{in}} + \left[\sqrt{\frac{G_3 T}{2}} - \sqrt{\frac{G_3(1-T)}{4}}\right]\hat{a}_{\text{EPR1}} \\
&\quad - \sqrt{\frac{G_3 - 1}{2}}\hat{b}_{\text{EPR1}}^\dagger - \sqrt{\frac{G_3(1-T)}{2}}\hat{a}_{\text{EPR2}} + \sqrt{\frac{G_3 - 1}{2}}\hat{b}_{\text{EPR2}}^\dagger \\
&\quad + \left[\sqrt{\frac{G_3 T}{2}} - \sqrt{\frac{G_3(1-T)}{4}} - \sqrt{\frac{G_3 - 1}{2}}\right]\delta N_1 \\
&\quad + \left[-\sqrt{\frac{G_3(1-T)}{2}} + \sqrt{\frac{G_3 - 1}{2}}\right]\delta N_2.
\end{aligned} \tag{S19}
$$

Considering $G_3 = 7 - 4\sqrt{2}$ and $T = \left(4\sqrt{2} + 7\right)/17$, the amplitude quadrature and phase quadrature of this output state $\hat{a}_{\text{out}}$ can be expressed as

$$
\begin{aligned}
\hat{X}_{\text{out}} &= \hat{X}_{\text{in}} + g_{1x}\left(\hat{X}_{\hat{a}_{\text{EPR1}}} - \hat{X}_{\hat{b}_{\text{EPR1}}}\right) + g_{1x}\left(\hat{X}_{\hat{b}_{\text{EPR2}}} - \hat{X}_{\hat{a}_{\text{EPR2}}}\right), \\
\hat{Y}_{\text{out}} &= \hat{Y}_{\text{in}} + g_{1y}\left(\hat{Y}_{\hat{a}_{\text{EPR1}}} + \hat{Y}_{\hat{b}_{\text{EPR1}}}\right) - g_{1y}\left(\hat{Y}_{\hat{a}_{\text{EPR2}}} + \hat{Y}_{\hat{b}_{\text{EPR2}}}\right),
\end{aligned} \tag{S20}
$$

where $g_{1y} = g_{1x} = \sqrt{2} - 1$. The corresponding quadrature variances can be given by

$$
\Delta^2\hat{X}_{\text{out}} = \Delta^2\hat{Y}_{\text{out}} = 1 + (3 - 2\sqrt{2})\left[4(G_1 + G_2) - 4\sqrt{G_1(G_1 - 1)} - 4\sqrt{G_2(G_2 - 1)} - 4\right]. \tag{S21}
$$

The fidelity of {1, 2, 4} reconstruction structure can be calculated as

$$
F_{\{1,2,4\}} = \frac{1}{1 + (3 - 2\sqrt{2})\left[2(G_1 + G_2) - 2\sqrt{G_1(G_1 - 1)} - 2\sqrt{G_2(G_2 - 1)} - 2\right]}. \tag{S22}
$$

When $G_1 = 1$, $G_2 = 1$, $G_3 = 7 - 4\sqrt{2}$ and $T = \left(4\sqrt{2} + 7\right)/17$, the classical fidelity of {1, 2, 4} reconstruction structure can be calculated as $F_{\{1,2,4\}}^{\text{clas}} = 1/(7 - 4\sqrt{2})$. The fidelities of {1, 2, 5}, {1, 3, 4}, and {1, 3, 5} reconstruction structures are equal to that of {1, 2, 4} structure.

For {2, 3, 5} reconstruction structure as shown in Fig. S1F, $\hat{a}_2$ and $\hat{a}_3$ are combined by a BS with a transmissivity of T. Then, with the help of $\hat{a}_5$, the output of this BS is amplified by a PIA, which can be given by

$$
\hat{a}_{\text{out}} = \sqrt{G_3}\left(\sqrt{T}\hat{a}_2 + \sqrt{1-T}\hat{a}_3 e^{i\theta_1}\right)e^{i\theta_2} + \sqrt{G_3 - 1}\hat{a}_5^\dagger, \tag{S23}
$$

where G_3 is the intensity gain of the PIA. θ_1 is the relative phase between $\hat{a}_2$ and $\hat{a}_3$, and θ_2 is the relative phase between $\hat{a}_5$ and the output of the BS. When $\theta_1 = 0$ and $\theta_2 = 0$, $\hat{a}_{\text{out}}$ can be given by

$$
\begin{aligned}
\hat{a}_{\text{out}} &= \sqrt{G_3}\left(\sqrt{T}\hat{a}_2 + \sqrt{1-T}\hat{a}_3\right) + \sqrt{G_3 - 1}\hat{a}_5^{\dagger} \\
&= \left[\sqrt{\frac{G_3 T}{4}} + \sqrt{\frac{G_3(1-T)}{4}}\right]\hat{a}_{\text{in}} - \left[\sqrt{\frac{G_3 T}{4}} + \sqrt{\frac{G_3(1-T)}{4}}\right]\hat{a}_{\text{EPR1}} \\
&\quad + \sqrt{\frac{G_3 - 1}{2}}\hat{b}_{\text{EPR1}}^{\dagger} - \left[\sqrt{\frac{G_3 T}{2}} - \sqrt{\frac{G_3(1-T)}{2}}\right]\hat{a}_{\text{EPR2}} + \sqrt{\frac{G_3 - 1}{2}}\hat{b}_{\text{EPR2}}^{\dagger} \\
&\quad + \left[\sqrt{\frac{G_3 - 1}{2}} - \sqrt{\frac{G_3 T}{4}} - \sqrt{\frac{G_3(1-T)}{4}}\right]\delta N_1 \\
&\quad + \left[\sqrt{\frac{G_3 - 1}{2}} - \sqrt{\frac{G_3 T}{2}} + \sqrt{\frac{G_3(1-T)}{2}}\right]\delta N_2.
\end{aligned}
\tag{S24}
$$

Considering $G_3 = 3$ and $T = \sqrt{2}/3 + 1/2$, the amplitude quadrature and phase quadrature of $\hat{a}_{\text{out}}$ can be expressed as

$$
\begin{aligned}
\hat{X}_{\text{out}} &= \hat{X}_{\text{in}} + \left(\hat{X}_{\hat{b}_{\text{EPR1}}} - \hat{X}_{\hat{a}_{\text{EPR1}}}\right) + \left(\hat{X}_{\hat{b}_{\text{EPR2}}} - \hat{X}_{\hat{a}_{\text{EPR2}}}\right), \\
\hat{Y}_{\text{out}} &= \hat{Y}_{\text{in}} - \left(\hat{Y}_{\hat{a}_{\text{EPR1}}} + \hat{Y}_{\hat{b}_{\text{EPR1}}}\right) - \left(\hat{Y}_{\hat{a}_{\text{EPR2}}} + \hat{Y}_{\hat{b}_{\text{EPR2}}}\right).
\end{aligned}
\tag{S25}
$$

The corresponding quadrature variances can be given by

$$
\Delta^2\hat{X}_{\text{out}} = \Delta^2\hat{Y}_{\text{out}} = 4(G_1 + G_2) - 4\sqrt{G_1(G_1 - 1)} - 4\sqrt{G_2(G_2 - 1)} - 3. \tag{S26}
$$

The fidelity of this {2, 3, 5} reconstruction structure can be calculated as

$$
F_{\{2,3,5\}} = \frac{1}{2(G_1 + G_2) - 2\sqrt{G_1(G_1 - 1)} - 2\sqrt{G_2(G_2 - 1)} - 1}. \tag{S27}
$$

When $G_1 = 1$, $G_2 = 1$, $G_3 = 3$ and $T = \sqrt{2}/3 + 1/2$, the classical fidelity of {2, 3, 5} reconstruction structure can be calculated as $F_{\{2,3,5\}}^{\text{clas}} = 1/3$. The fidelity of {2, 3, 4} reconstruction structure is equal to that of {2, 3, 5} reconstruction structure. By calculating the average classical fidelity of ten reconstruction structures, the average classical fidelity of (3, 5) threshold AOQSS protocol can be obtained, i.e., $F_{\text{lim}}^{\text{clas}} = 3/5\ (k/n)$. Similarly, when $G_1 \to \infty$ and $G_2 \to \infty$, the average fidelity of (3, 5) threshold deterministic AOQSS protocol can be given as $F_{\text{avg}} \to 1$. In other words, in principle, our AOQSS protocol can implement the perfect quantum state sharing of any continuous variable (CV) quantum state. Therefore, the average fidelity beats the corresponding classical limit $F_{\text{lim}}^{\text{clas}} = 3/5\ (k/n)$, indicating that the (3, 5) threshold deterministic AOQSS succeeds.

The detailed experimental setup for generating EPR entanglement array and constructing (3, 5) threshold AOQSS

The experimental scheme of the deterministic (3, 5) threshold AOQSS is shown in Fig. S2. As shown in Fig. S2A, our laser system is based on a Ti:sapphire laser with a frequency of about 1 GHz blue-detuned from the ^{85}Rb D1 line ($5S_{1/2}$, $F = 2 \to 5P_{1/2}$, 795 nm). The laser is divided into two by a polarization beam splitter (PBS1). We pass the vertically polarized one with a power of about 900 mW through two lenses to increase its waist size. Then, reflected by a Glan-Laser polarizer (GL), this vertically polarized beam is directed onto the 5×5 metalens array, producing a 5×5 pump array. In such a pump array, each of these pump spots has a waist of about 78.9 μm. The losses of metalens can decrease the powers of the 5×5 pump array. Because such pump power

decrease happens before the FWM process occurs, we can address the impact of such loss on entanglement by increasing the power of the laser seeded onto the 5×5 metalens array. Each of these pump spots can initiate a spontaneous FWM process in a hot ^{85}Rb vapor cell, wherein two pump photons convert into one signal beam photon (redshifted by about 3.04 GHz from the pump beam) and one idler beam photon (blueshifted by about 3.04 GHz from the pump beam) as shown in Fig. S2B. The absorption of the vapor cell will bring propagation loss. In our scheme, the propagation loss through the vapor cell is about 20%. Such loss can be decreased by changing the one-photon detuning, two-photon detuning, and the temperature of the vapor cell in the future. The temperature of ^{85}Rb vapor cell is about 118°C. For accurate temperature stabilization, a heating tape powered by an AC source is utilized to heat the ^{85}Rb vapor cell. We put a temperature sensor close to the ^{85}Rb vapor cell, which measures the temperature of the ^{85}Rb vapor cell. By utilizing a digital temperature controller (Omron E5EC-RR2ASM-820), switching on and off the heating tape can be realized, which depends on the difference between actual temperature and set temperature. In this way, we can maintain the temperature of the ^{85}Rb vapor cell at 118°C with an accuracy of 0.01°C. The resulting signal beam and idler beams exhibit EPR entanglement, generating a 5×5 EPR entanglement array. For constructing (3, 5) threshold AOQSS, two pairs of EPR entanglements ($\hat{a}_{\text{EPR1}}$ and $\hat{b}_{\text{EPR1}}$, $\hat{a}_{\text{EPR2}}$ and $\hat{b}_{\text{EPR2}}$) from 5×5 EPR entanglement array are utilized. Then, we further divide the horizontally polarized one from PBS1 into two beams by PBS2. The weak one passes through an acousto-optic modulator (AOM) to obtain the secret coherent state $\hat{a}_{\text{in}}$ which is redshifted by 3.04 GHz from the pump beam. The quadrature noise level of the secret state is almost equal to that of the vacuum state. After that, $\hat{a}_{\text{in}}$ and $\hat{a}_{\text{EPR1}}$ are combined by a 50:50 BS1. One output of BS1 denotes $\hat{a}_1$. The other output of BS1 and $\hat{a}_{\text{EPR2}}$ are combined by another 50:50 BS2, obtaining $\hat{a}_2$ and $\hat{a}_3$. $\hat{a}_4$ and $\hat{a}_5$ are obtained by combining $\hat{b}_{\text{EPR1}}$ and $\hat{b}_{\text{EPR2}}$ with 50:50 BS3. Then, $\hat{a}_1$ to $\hat{a}_5$ are sent to five users, respectively.

In (3, 5) threshold AOQSS, there are ten reconstruction protocols. We send these shares into different reconstruction boxes, which are indicated by Figs. S2C to S2G. In {1, 2, 3} reconstruction structure shown in Fig. S2C, $\hat{a}_2$ and $\hat{a}_3$ are combined with 50:50 BS4. The piezo-electric transducer (PZT) placed in the path of $\hat{a}_2$ is used to change the relative phase between $\hat{a}_2$ and $\hat{a}_3$. After locking the relative phase (phase stabilization) between $\hat{a}_2$ and $\hat{a}_3$ with an MCU, the BS4 has one bright output and one vacuum output. Then, the bright output and $\hat{a}_1$ are combined with 50:50 BS5. By the same phase-locking, the bright output of BS5 is the recovered state of the {1, 2, 3} reconstruction structure. In {1, 4, 5} reconstruction structure shown in Fig. S2D, $\hat{a}_4$ and $\hat{a}_5$ are combined by BS6 with a transmissivity of 1/2. A PZT placed in the path of $\hat{a}_4$ is used to scan the relative phase between $\hat{a}_4$ and $\hat{a}_5$. To recover the secret state, $\hat{a}_1$ should be amplified with the help of output beam from BS6 in a PIA based on the FWM process. The strong one from PBS2 serves as the pump for the PIA. Reflected by a GL, $\hat{a}_1$, output beam from BS6, and pump beam are crossed at the center of the 12-mm long ^{85}Rb vapor cell. When the intensity gain of the PIA is about 2, the secret state can be reconstructed. As shown in Fig. S2E, the {2, 4, 5} and {3, 4, 5} reconstruction structures are similar to {1, 4, 5} reconstruction structure. In {1, 2, 4} reconstruction structure shown in Fig. S2F, $\hat{a}_1$ and $\hat{a}_2$ are combined by BS8 with a transmissivity of about 0.74. Because $\hat{a}_1$ and $\hat{a}_2$ are bright, the relative phase between $\hat{a}_1$ and $\hat{a}_2$ should be locked with MCU. Reflected by a GL, $\hat{a}_4$, output beam from BS8, and pump beam are crossed at the center of the 12-mm long ^{85}Rb vapor cell. When the intensity gain of the PIA is about 1.34, the secret state can be reconstructed. As shown in Figs. S2F and S2G, the {1, 2, 5}, {1, 3, 4}, {1, 3, 5}, {2, 3, 4}, and {2, 3, 5} reconstruction structures are similar to {1, 2, 4} reconstruction structure.

To check the performance of (3, 5) threshold AOQSS, the recovered state $\hat{a}_{\text{out}}$ is measured by a balanced homodyne detection (BHD). We obtain the local oscillator (LO) by setting up a similar setup which is a few millimeters above the current beams. The powers of local oscillator beams are about 800 μW. The transimpedance gain and the quantum efficiency of the balanced detector are 10^5 V/A and 97% (high enough for detection), respectively. The amplitude (phase) quadrature variance of $\hat{a}_{\text{out}}$ is analyzed by a spectrum analyzer (SA). The SA is set to a 1 MHz resolution bandwidth (RBW) and a 100 Hz video bandwidth (VBW).

Metalens design for compact large scale EPR states generation

Figure S3A shows the schematic working process of metalens array assisted large scale EPR states generation process. To select each pair (for example, EPR1 and EPR2) in the EPR entanglement array and optimize the coupling of our scheme in dealer protocol and reconstruction part (especially in PIA), a bright signal beam is required to assist the alignment and selection. The angle θ between bright signal beam and pump beam is about 2 to 10 mrad due to phase-match conditions of FWM process *(48)*. Without loss of generality, here the angle θ is set as 10 mrad. The pump beam, shown in light red, is normally incident to the metalens array, and meanwhile, the signal beam, shown in light yellow, is incident to the array with an angle of 10 mrad to the pump beam. The focus of the pump beam through the lower metalens and that of the signal beam through the upper metalens should overlap with each other at the center of the ^{85}Rb vapor cell to ensure the amplification gain of FWM process. To meet this requirement, the focal length of the metalenses is designed to be 3 cm with a metalens array period of 300×300 μm^2. In this way, we can obtain the naturally separated signal array and idler array generated from FWM processes as shown in Figs. 1B, 2A, and 2B of the main text. Then, according to the corresponding relationship between signal beam and idler beam shown in Fig. 1B of the main text, we can select the corresponding signal and idler beam of each pair (for example, EPR1 and EPR2) in the EPR entanglement array by utilizing several D-shaped pickoff mirrors. After completing the alignment and selection, we block the injected bright signal beam in generating 5×5 EPR entanglement array.

The metalenses are designed according to the phase requirement of the focal lens

$$\varphi(R,\lambda) = -\left[2\pi\left(\sqrt{R^2+f^2}-f\right)\right]\frac{1}{\lambda}. \tag{S28}$$

Here, R corresponds to the position in the metalens, λ is the working wavelength 795 nm. The target f is the focal length (3 cm). However, the numerical aperture (NA) of the lens is very low, and the realistic focal length deviates from that determined by the above formula substantially. To obtain the exact focal length, we performed a numerical calculation and found that the realistic focal length reaches approximately 3 cm when f is set to about 7 cm in the above formula. Figure S3B shows the phase distribution of the metalens when f=7 cm. Figure S3C depicts the corresponding calculated intensity distribution, with the focal length being about 3 cm as required.

To be noticed, the phase range of the lens spans only about $\Delta\varphi = 0.4\,\pi$ across the diameter. This poses a substantial challenge to traditional optical devices, including microlens array devices. Assuming the lens is made of glass, the refractive index difference between the glass and air is approximately

$$\Delta n = n_{\text{glass}} - n_{\text{air}} = 0.5. \tag{S29}$$

To achieve such a phase distribution, the thickness difference range is

$$\Delta l = \Delta\varphi/(\Delta n \cdot k_0) \tag{S30}$$

Which is about 300 nm. Precisely controlling the thickness variation in such a range for traditional refractive optical devices is extremely challenging. However, it is relatively easy to achieve such a phase difference by controlling the lateral dimensions of nanostructures with metasurfaces.

Here, the metalenses are designed with TiO_2 nanocylinders to achieve the required phase modulation. The TiO_2 has high refractive index and no absorption in the spectral range from 400 nm to 1000 nm, as shown in Figure S4. The height of nanocylinders is 1200 nm, and period of the nanocylinders array is 360 nm. Numerical simulations were performed using the commercial software Lumerical FDTD to determine the transmission performance of the unit cell of the TiO_2 nanocylinder array. Subsequently, 24 different nanocylinders with varying radii were selected to provide phase modulation in the range of 0-345 degrees. Table S1 lists the geometric information of all the nanocylinders along with their corresponding phase modulations. Nanocylinders were chosen from this table to meet the phase requirements of the metalens.

The nanocylinders achieve a phase accuracy of 15° with a radius difference in the range of 3 to 9 nm, as shown in Table S1. Notably, the accuracy of current nanofabrication technology surpasses this requirement, enabling even better phase accuracy. Consequently, there is potential to achieve more precise phase control and to realize smaller metalens arrays, leading to more compact designs.

Characterization of the metalens array

Figure S6A shows the scanning electron microscope (SEM) image of the fabricated sample, while Fig. S6B presents the optical microscopy image of a 3×3 metalens area of the sample. Figure S6C depicts the image of the focal plane of the metalens array under the pump laser with the wavelength of 795nm. To characterize the metalens array sample, we built a microscopy system as shown in Fig. S6D. The pump beam modulated by the metasurface sample is collected by an objective lens (10×, NA = 0.26), and the light intensity information is recorded by the charge coupled device (CCD) camera. The metasurface sample is mounted on a high-precision three-dimensional (3D) displacement platform. The truncated images of planes at various distances from the sample are recorded by the CCD camera. Finally, the intensity profile of the focusing in the x-z plane can be reconstructed by these recorded images. Figure S6E shows the typical focusing profile of the metalens array sample, with a focusing efficiency of 47.3 ± 3.3% as shown in Fig. S6F. Additionally, the measured focal length is 2.99 ± 0.03 cm, with the full width at half maximum (FWHM) of the focus at 78.9 ± 2.3 μm, as presented in Figure S6G.

Fabrication tolerance

The main fabrication imperfections arise from variations in nanostructure height, inaccurate dimensions of the nano-cylinders, and non-vertical etching. To investigate the tolerance of the metalens to these imperfections, we performed numerical simulations based on experimental data of our fabrication process. Below is the detailed discussion of these fabrication imperfections.

1. Height Variations: For the height of the TiO_2 nanostructures, the thickness of the deposited film can be well controlled at 1200 ± 20 nm. Figures S7A and S7B show the simulated results of the transmittance and phase evolution under such variations, respectively. Simulations indicate that within this range, the transmittance of the metasurface unit cells remains above 90% as shown in Fig. S7A, with negligible impact on transmission efficiency. Although height variations affect the

phase of the nanocylinders, the relative phase changes are nearly consistent, minimizing the effect on focusing performance. To evaluate the focusing performance of the designed metalens under height imperfections, we conducted calculations based on the simulated phase evolutions. The results show that a height of 1200 - 20 nm results in a focal length of 3.06 cm and an FWHM of 76.1 μm, while a height of 1200 + 20 nm results in a focal length of 2.96 cm and an FWHM of 77.2 μm. These minor changes in focal length do not substantially impact the FWM process.

2. Dimension Variations: During fabrication, the dimension of the nanocylinders may deviate from the designed value, causing some phase variation from the design data. Nevertheless, within a single sample, the size variations of the nanoposts are nearly consistent. In our fabrication, the diameter deviation can be well controlled to ± 10 nm. Figures S7C and S7D show the simulated results of the transmittance and phase evolution under such variations, respectively. The transmittances do not show substantial variations as well. Meanwhile, the focusing performances are calculated based on the simulated phase evolution with the originally designed metalens arrangement. The calculated focusing performance reveals that a 10 nm increase in diameter will result in a focal length of 2.96 cm and an FWHM of 74.9 μm, while a 10 nm decrease leads to a focal length of 3.09 cm and an FWHM of 77.6 μm, which are close to the performance without imperfection. Such minor variations have negligible effects on the FWM process.

3. Sidewall Imperfections: Non-vertical sidewalls may arise from imperfect etching during the fabrication. In our fabrication process, a pre-etching test step is employed to control the etching angle, ensuring sidewalls within angles between about 88° and 90°. Figures S7E and S7F show the simulated transmittance and phase evolution of the nanoposts in these conditions. The transmittance remains above about 95% for both scenarios. The sidewall imperfections introduce phase variations at the case of 88°, which will result in a focal length of 3.15 cm for the metalens. However, since this change uniformly affects all metalenses in the sample, the focal length across the array remains consistent in this situation. Meanwhile, the FWHM is 80.6 μm under the 88° etching imperfection, which still represents a good focusing performance. In this case, a slight adjustment of the seeded signal beam angle can compensate for the variation, ensuring no substantial impact on the FWM process.

Overall, fabrication imperfections introduce some variation in focusing. The focusing efficiency, however, remains largely unaffected. Imperfections primarily influence the phase modulation of the micro-nanostructures, leading to changes in focal length. Nevertheless, in our fabrication process, these imperfections are consistent across the entire sample. Consequently, even if these imperfections cause variations in focal length, they can be mitigated through slight adjustments to the seeded signal beam angle. These results demonstrate that the metalenses exhibit strong robustness to the typical imperfections inherent in our fabrication process.

Scalability of the metalens-array-based EPR entangled states

In this work, we demonstrate the generation of large-scale EPR entangled states using a 5×5 metalens array. By incorporating additional metalenses into the array, the network scale can be substantially expanded (*31*). However, scaling up the metalens array introduces challenges related to uniformity and fabrication stability. Fortunately, these issues can be effectively addressed through advancements in both research laboratories and high-throughput mass production techniques. Notably, centimeter-scale metalenses with exceptional performance in the near-

infrared and visible ranges have already been fabricated using industrial methods such as high energy e-beam lithography, deep ultraviolet lithography, and nanoimprinting, even on 8-inch wafers (*49, 57, 58*). Utilizing these advanced fabrication technologies, metasurface arrays can be greatly expanded to achieve larger-scale EPR entangled states, further supporting the demands of large-scale quantum information networks.

Evidence to support the presence of the entanglement array

The sufficient criterion of the entanglement can be characterized by inseparability $I_{\hat{a},\hat{b}}$ where $I_{\hat{a},\hat{b}}$ = $\mathrm{Var}(\hat{X}_{\hat{a}} - \hat{X}_{\hat{b}}) + \mathrm{Var}(\hat{Y}_{\hat{a}} + \hat{Y}_{\hat{b}})$. If $I_{\hat{a},\hat{b}} < 4$, two optical fields $\hat{a}$ and $\hat{b}$ are entangled. As shown in Fig. S8, the inseparabilities for the 5×5 entanglement array are all less than 4, demonstrating the existence of 25 entanglement pairs in our scheme.

State-of-the-art methods for CV EPR entanglement generation and AOQSS

Table S2 compares our metalens-based EPR entanglement array with other state-of-the-art methods for CV EPR entanglement generation based on different multiplexed degrees of freedom. In particular, it includes the key metrics such as the number of EPR pairs, mode natural separation, multiplexing, maximum entanglement squeezing, generation rates, and long-term stability. The number of EPR pairs generated in our scheme surpasses those of other works (*12*, *59-61*). More importantly, compared with these works (*12*, *59-61*), the optical modes of our metalens-based EPR entanglement array are naturally separated. This feature guarantees that our EPR entanglement array can be directly applied in constructing multi-user quantum communication network. In terms of generation rates, the CV EPR entanglement generated by both our metalens-based approach and other state-of-the-art methods are all deterministic. In terms of long-term stability, the entanglement stability is mainly determined by the temperature stability of nonlinear medium and the stability of the laser power and frequency. Therefore, our metalens-based approach has the same level of performance as other state-of-the-art methods (*12*, *59-61*) in terms of generation rates and long-term stability.

In addition, compared with a three-user AOQSS constructed in the previous experiment (*44*), we have constructed a five-user AOQSS network in this work, which is currently the largest AOQSS network in the CV regime.

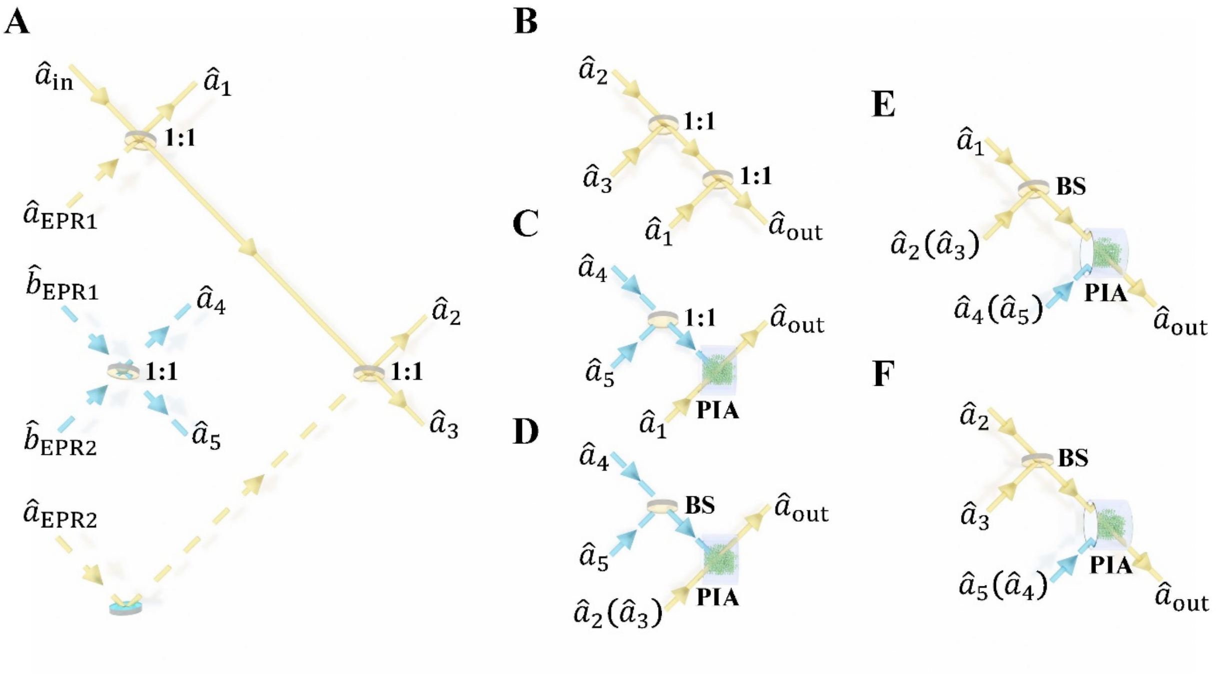

Fig. S1.

The structure of (3, 5) threshold deterministic all-optical quantum state sharing (AOQSS).
(A) The dealer protocol. $\hat{a}_{\text{EPR1}}$ and $\hat{b}_{\text{EPR1}}$ ($\hat{a}_{\text{EPR2}}$ and $\hat{b}_{\text{EPR2}}$), the annihilation operators associated with Einstein-Podolsky-Rosen (EPR) entangled source EPR1 (EPR2); 1:1, 50:50 beam splitter; $\hat{a}_{\text{in}}$, the annihilation operator of the secret coherent state; $\hat{a}_1$, $\hat{a}_2$, $\hat{a}_3$, $\hat{a}_4$, and $\hat{a}_5$, the annihilation operators of five shares. (B) {1, 2, 3} reconstruction structure. (C) {1, 4, 5} reconstruction structure. (D) {2, 4, 5} ({3, 4, 5}) reconstruction structure. (E) {1, 2, 4} ({1, 2, 5}, {1, 3, 4}, {1, 3, 5}) reconstruction structure. (F) {2, 3, 5} ({2, 3, 4}) reconstruction structure. BS, beam splitter. $\hat{a}_{\text{out}}$, the annihilation operator of the output reconstructed state; PIA, phase-insensitive amplifier.

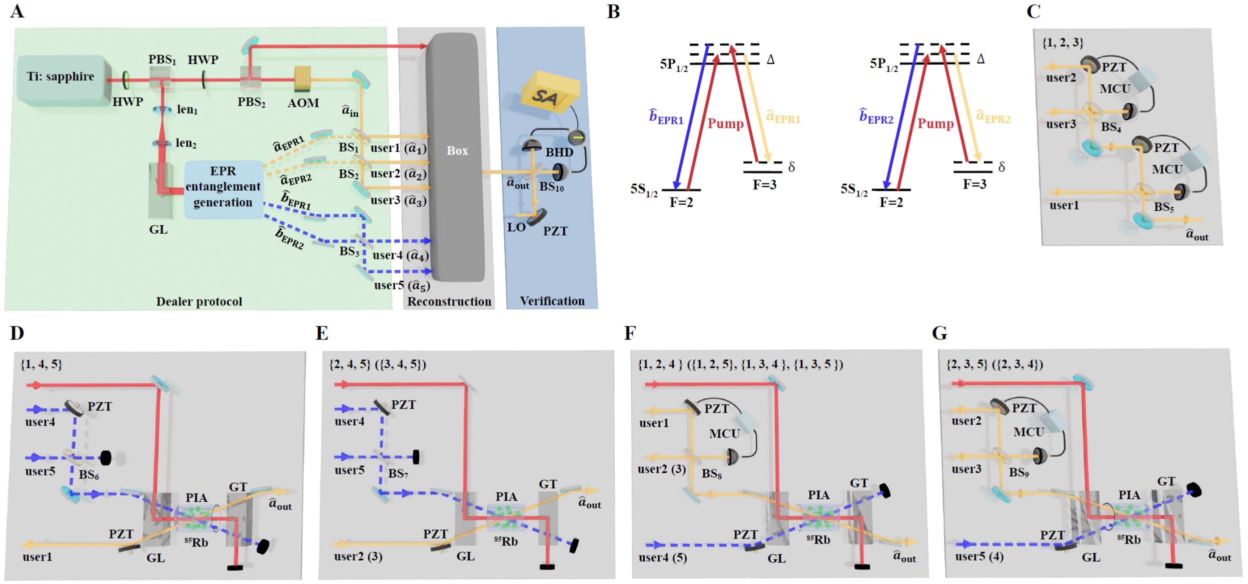

Fig. S2.

The detailed experimental scheme of (3, 5) threshold deterministic all-optical quantum state sharing (AOQSS). (A) The detailed experimental setup. (B) Energy level diagram of ^{85}Rb D1 line. Δ, one-photon detuning; δ, two-photon detuning. (C) to (G) different reconstruction structures. HWP, half-wave plate; PBS, polarization beam splitter; GL, Glan–laser polarizer; AOM, acousto-optic modulator; BS, beam splitter; PZT, piezo-electric transducer; MCU, micro-control unit; GT, Glan–Thompson polarizer; LO, local oscillator; BHD balanced homodyne detection; SA, spectrum analyzer; $\hat{a}_{\text{in}}$, the annihilation operator associated with input coherent state; $\hat{a}_{\text{out}}$, the annihilation operator associated with output state; $\hat{a}_1$ to $\hat{a}_5$, the annihilation operators associated with five shares held by user1 to user5, respectively.

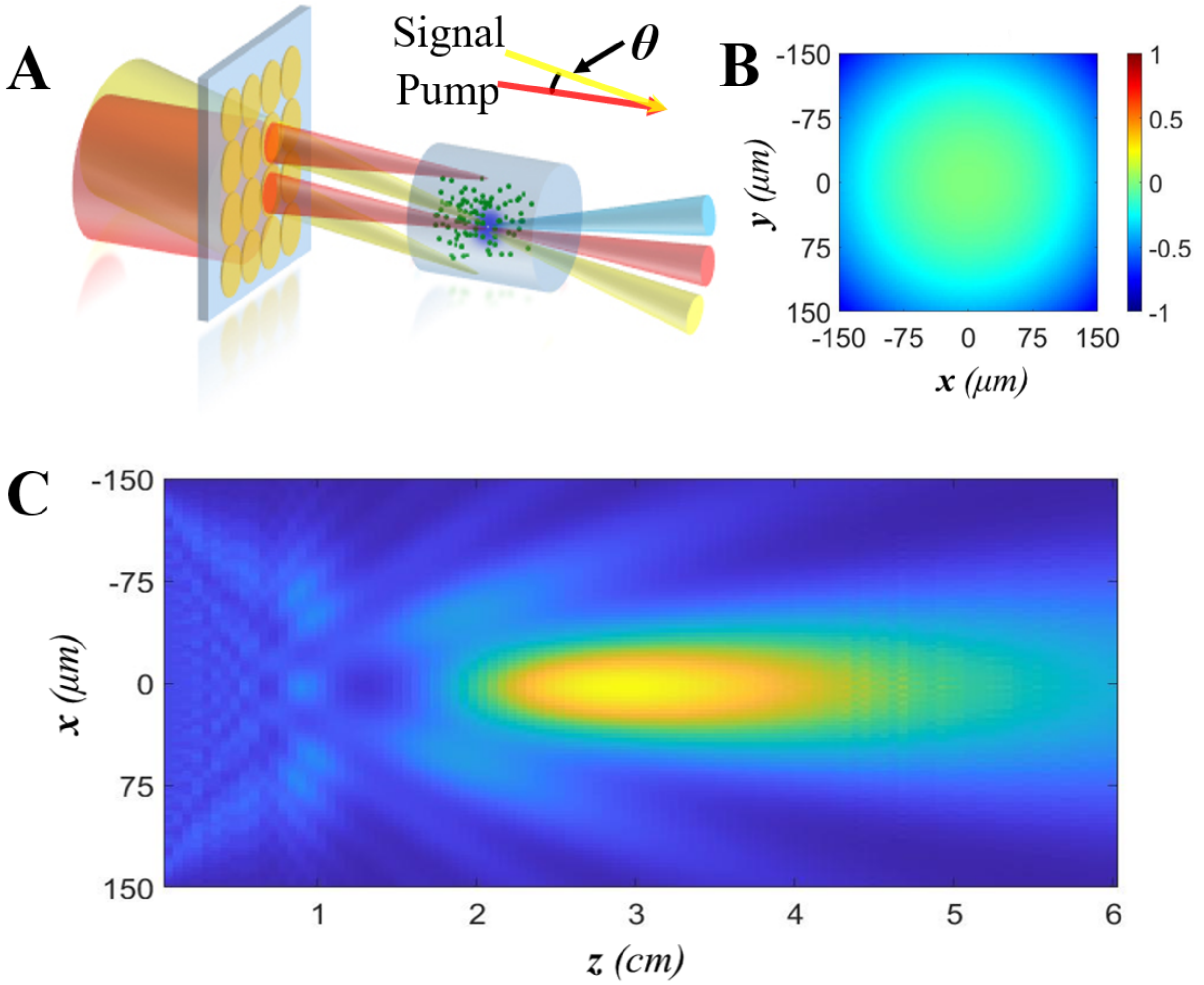

Fig. S3.

Metalens design for compact large scale Einstein-Podolsky-Rosen (EPR) states generation. (A) The schematic figure of the metalens array assists four-wave mixing (FWM) process to generate large scale EPR states. (B) The phase distribution of the designed metalens. (C) The corresponding calculated intensity distribution of the focusing realized by the metalens.

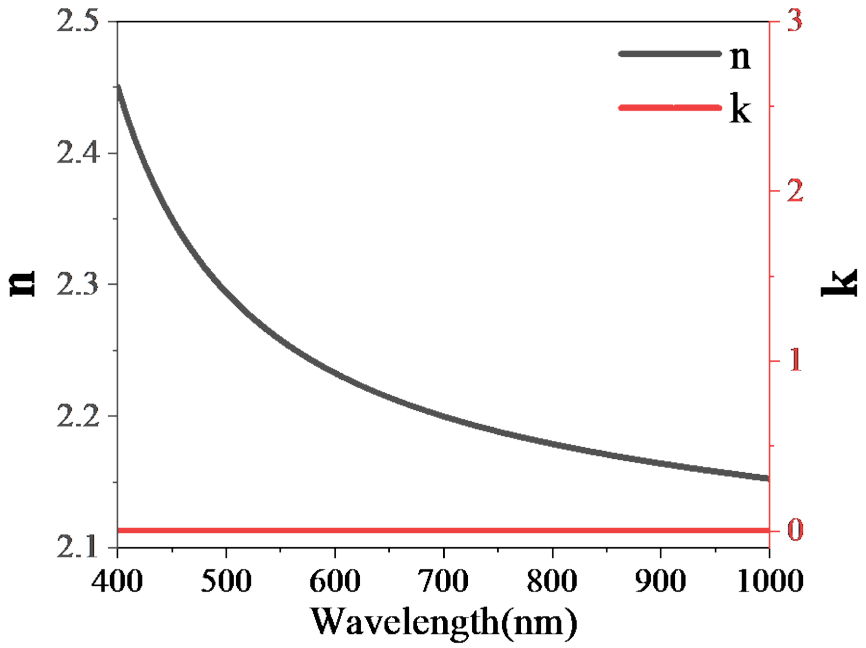

Fig. S4.

Refractive index and absorption of TiO_2.

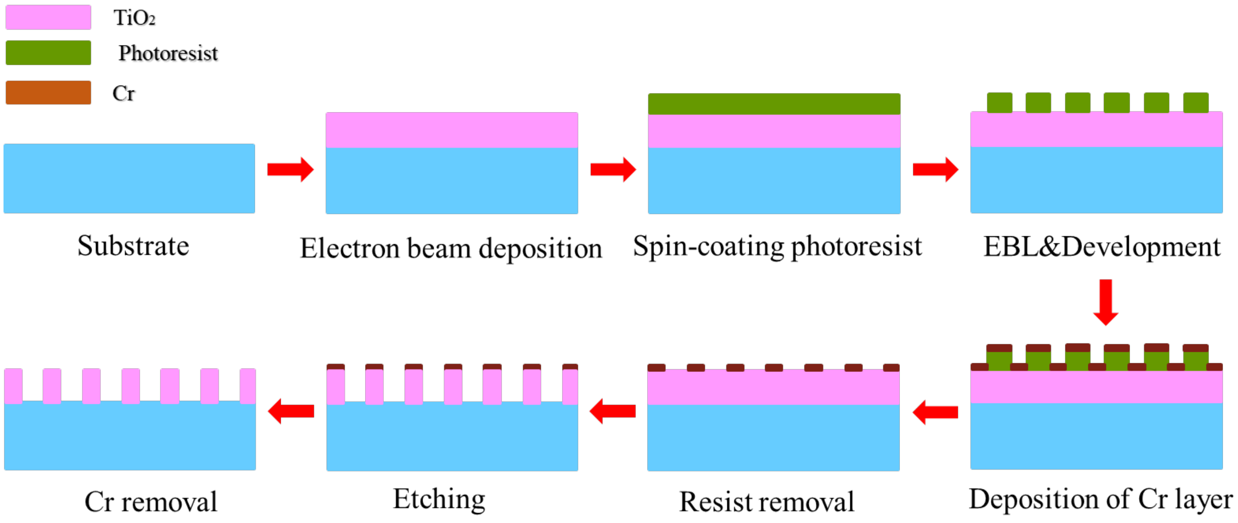

Fig. S5.

The schematic of the fabrication process.

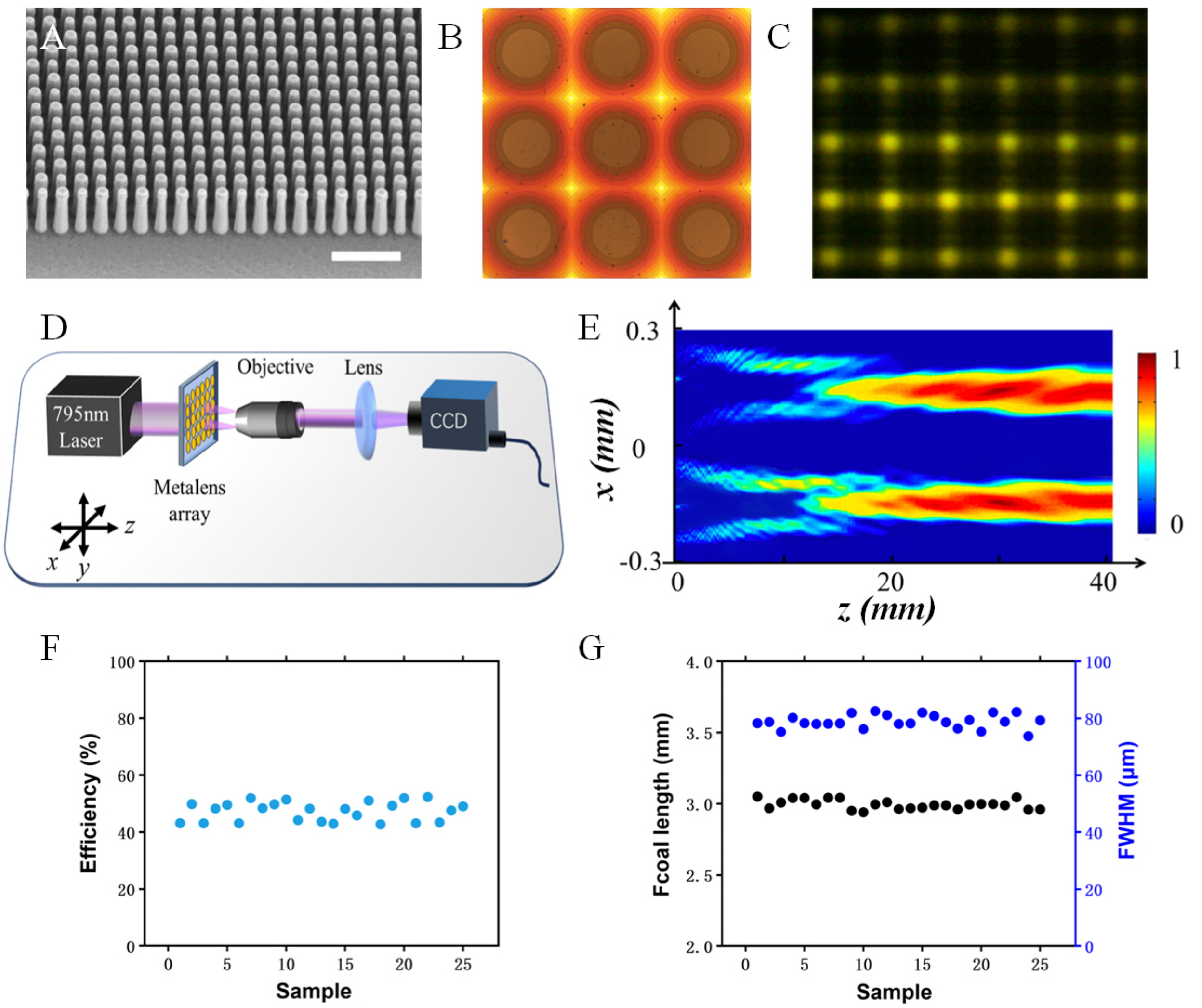

Fig. S6.

The characterization of the fabricated metasurface sample. (A) scanning electron microscope (SEM) image. (B) The microscopy image. (C) The image of the focal plane (x-y plane) under the pump beam. (D) The measurement system for the optical property of the metasurface. (E) The intensity distribution in x-z plane of the focusing beam. (F) The measured focusing efficiencies of the metalenses. (G) The focal length and the full width at half maximum (FWHM) of the focal spots.

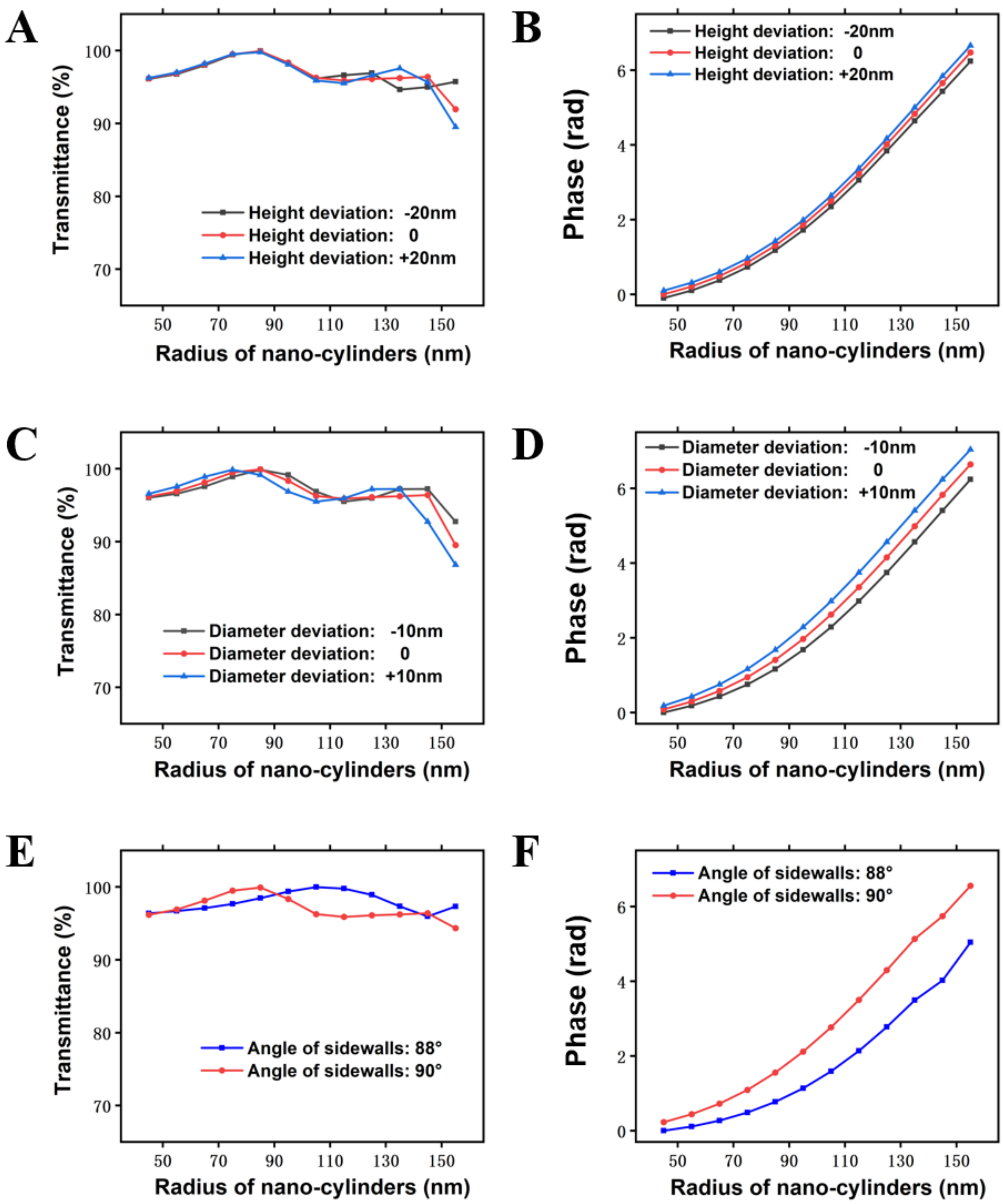

Fig. S7.

The impact of the fabrication imperfections. The simulated results of the transmittance variations of the nanocylinders under different fabrication imperfections, including variations in the height (A), diameter (C) and sidewall angle (E). The simulated phase variations caused by these imperfections correspond to variations in height (B), diameter (D), and sidewall angle (F).

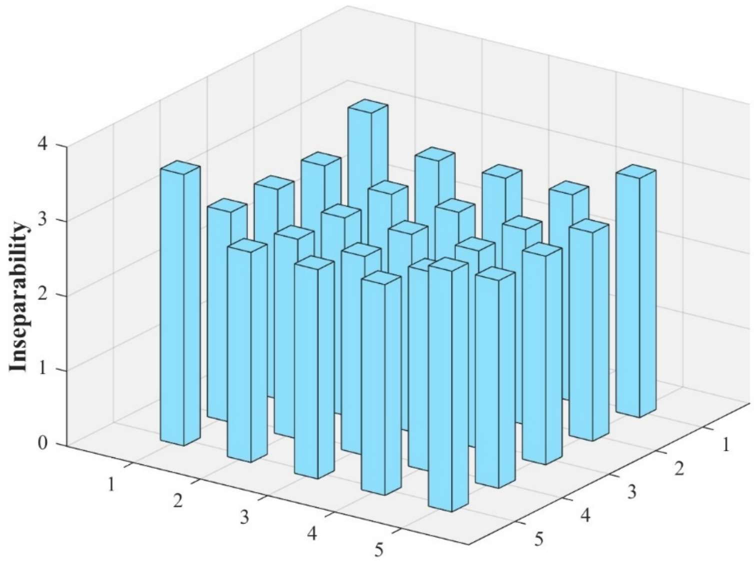

Fig. S8.
The inseparabilities of 5×5 entanglement array.

Number	Radius (nm)	Phase (degree)	Number	Radius (nm)	Phase (degree)
1	57	0	13	117	180
2	66	15	14	121	195
3	73	30	15	124	210
4	80	45	16	127	225
5	85	60	17	131	240
6	90	75	18	134	255
7	95	90	19	137	270
8	99	105	20	140	285
9	103	120	21	143	300
10	107	135	22	147	315
11	111	150	23	150	330
12	114	165	24	153	345

Table S1. 24 unit elements of metalens corresponding to 24 different phases.

**Number of EPR pairs**	**Mode natural separation**	**Multiplexing**	**Maximum entanglement squeezing**	**Generation rates**	**Long-term stability**	**Reference**
3	No	Temporal mode multiplexing	2.56 dB	Deterministic	Same level	Phys. Rev. Lett. 124, 213603 (2020) (*59*)
4	No	Frequency multiplexing	8.0 dB	Deterministic	Same level	Phys. Rev. Lett. 125, 070502 (2020) (*60*)
11	No	Orbital angular momentum multiplexing	3.0 dB	Deterministic	Same level	Nat. Commun. 11, 3875 (2020) (*12*)
20	No	Frequency multiplexing	1.6 dB	Deterministic	Same level	Nat. Commun. 12, 4781 (2021) (*61*)
**25**	**Yes**	Spatial multiplexing	1.89 dB	Deterministic	Same level	This work

Table S2. State-of-the-art methods for continuous variable (CV) Einstein-Podolsky-Rosen (EPR) entanglement generation.

REFERENCES AND NOTES

1. A. Galindo, M. A. Martín-Delgado, Information and computation: Classical and quantum aspects. *Rev. Mod. Phys.* **74**, 347–423 (2002).

2. J.-W. Pan, Z.-B. Chen, C.-Y. Lu, H. Weinfurter, A. Zeilinger, M. Żukowski, Multiphoton entanglement and interferometry. *Rev. Mod. Phys.* **84**, 777–838 (2012).

3. S. L. Braunstein, P. Van Loock, Quantum information with continuous variables. *Rev. Mod. Phys.***77**, 513–577 (2005).

4. C. H. Bennett, G. Brassard, C. Crépeau, R. Jozsa, A. Peres, W. K. Wootters, Teleporting an unknown quantum state via dual classical and Einstein-Podolsky-Rosen channels. *Phys. Rev. Lett.* **70**, 1895–1899 (1993).

5. D. Bouwmeester, J.-W. Pan, K. Mattle, M. Eibl, H. Weinfurter, A. Zeilinger, Experimental quantum teleportation. *Nature* **390**, 575–579 (1997).

6. S. L. Braunstein, H. J. Kimble, Teleportation of continuous quantum variables. *Phys. Rev. Lett.* **80**, 869–872 (1998).

7. A. Furusawa, J. L. Sørensen, S. L. Braunstein, C. A. Fuchs, H. J. Kimble, E. S. Polzik, Unconditional quantum teleportation. *Science* **282**, 706–709 (1998).

8. M. Riebe, H. Häffner, C. F. Roos, W. Hänsel, J. Benhelm, G. P. T. Lancaster, T. W. Körber, C. Becher, F. Schmidt-Kaler, D. F. V. James, R. Blatt, Deterministic quantum teleportation with atoms. *Nature* **429**, 734–737 (2004).

9. S. Olmschenk, D. N. Matsukevich, P. Maunz, D. Hayes, L.-M. Duan, C. Monroe, Quantum teleportation between distant matter qubits. *Science* **323**, 486–489 (2009).

10. M. Huo, J. Qin, J. Cheng, Z. Yan, Z. Qin, X. Su, X. Jia, C. Xie, K. Peng, Deterministic quantum teleportation through fiber channels. *Sci. Adv.* **4**, eaas9401 (2018).

11. T. C. Ralph, All-optical quantum teleportation. *Opt. Lett.* **24**, 348–350 (1999).

12. S. Liu, Y. Lou, J. Jing, Orbital angular momentum multiplexed deterministic all-optical quantum teleportation. *Nat. Commun.* **11**, 3875 (2020).

13. V. Scarani, H. Bechmann-Pasquinucci, N. J. Cerf, M. Dušek, N. Lütkenhaus, M. Peev, The security of practical quantum key distribution. *Rev. Mod. Phys.* **81**, 1301–1350 (2009).

14. F. Xu, X. Ma, Q. Zhang, H.-K. Lo, J.-W. Pan, Secure quantum key distribution with realistic devices. *Rev. Mod. Phys.* **92**, 025002 (2020).

15. V. Bužek, M. Hillery, Quantum copying: Beyond the no-cloning theorem. *Phys. Rev. A* **54**, 1844–1852 (1996).

16. V. Scarani, S. Iblisdir, N. Gisin, A. Acín, Quantum cloning. *Rev. Mod. Phys.* **77**, 1225–1256 (2005).

17. A. Lamas-Linares, C. Simon, J. C. Howell, D. Bouwmeester, Experimental quantum cloning of single photons. *Science* **296**, 712–714 (2002).

18. U. L. Andersen, V. Josse, G. Leuchs, Unconditional quantum cloning of coherent states with linear optics. *Phys. Rev. Lett.* **94**, 240503 (2005).

19. S. Liu, Y. Lou, Y. Chen, J. Jing, All-optical optimal n-to-m quantum cloning of coherent states. *Phys. Rev. Lett.* **126**, 060503 (2021).

20. A. Einstein, B. Podolsky, N. Rosen, Can quantum-mechanical description of physical reality be considered complete? *Phys. Rev.* **47**, 777–780 (1935).

21. N. Yu, F. Capasso, Flat optics with designer metasurfaces. *Nat. Mater.* **13**, 139–150 (2014).

22. N. Yu, P. Genevet, M. A. Kats, F. Aieta, J.-P. Tetienne, F. Capasso, Z. Gaburro, Light propagation with phase discontinuities: Generalized laws of reflection and refraction. *Science* **334**, 333–337 (2011).

23. X. Ni, N. K. Emani, A. V. Kildishev, A. Boltasseva, V. M. Shalaev, Broadband light bending with plasmonic nanoantennas. *Science* **335**, 427–427 (2012).

24. Q. He, S. Sun, L. Zhou, Tunable/reconfigurable metasurfaces: Physics and applications. *Research* **2019**, 1849272 (2019).

25. A. V. Kildishev, A. Boltasseva, V. M. Shalaev, Planar photonics with metasurfaces. *Science* **339**, 1232009 (2013).

26. H.-T. Chen, A. J. Taylor, N. Yu, A review of metasurfaces: Physics and applications. *Rep. Prog. Phys.* **79**, 076401 (2016).

27. Y. Gao, Z. Wang, Y. Jiang, R. Peng, Z. Wang, D. Qi, R. Fan, W. Tang, M. Wang, Multichannel distribution and transformation of entangled photons with dielectric metasurfaces. *Phys. Rev. Lett.* **129**, 023601 (2022).

28. T. Stav, A. Faerman, E. Maguid, D. Oren, V. Kleiner, E. Hasman, M. Segev, Quantum entanglement of the spin and orbital angular momentum of photons using metamaterials. *Science* **361**, 1101–1104 (2018).

29. K. Wang, J. G. Titchener, S. S. Kruk, L. Xu, H.-P. Chung, M. Parry, I. I. Kravchenko, Y.-H. Chen, A. S. Solntsev, Y. S. Kivshar, D. N. Neshev, A. A. Sukhorukov, Quantum metasurface for multiphoton interference and state reconstruction. *Science* **361**, 1104–1108 (2018).

30. P. Georgi, M. Massaro, K.-H. Luo, B. Sain, N. Montaut, H. Herrmann, T. Weiss, G. Li, C. Silberhorn, T. Zentgraf, Metasurface interferometry toward quantum sensors. *Light Sci Appl* **8**, 70 (2019).

31. L. Li, Z. Liu, X. Ren, S. Wang, V.-C. Su, M.-K. Chen, C. H. Chu, H. Y. Kuo, B. Liu, W. Zang, G. Guo, L. Zhang, Z. Wang, S. Zhu, D. P. Tsai, Metalens-array–based high-dimensional and multiphoton quantum source. *Science* **368**, 1487–1490 (2020).

32. J. Zhou, S. Liu, H. Qian, Y. Li, H. Luo, S. Wen, Z. Zhou, G. Guo, B. Shi, Z. Liu, Metasurface enabled quantum edge detection. *Sci. Adv.* **6**, eabc4385 (2020).

33. D. Zhang, Y. Chen, S. Gong, W. Wu, W. Cai, M. Ren, X. Ren, S. Zhang, G. Guo, Jingjun Xu, All-optical modulation of quantum states by nonlinear metasurface. *Light Sci Appl* **11**, 58 (2022).

34. T. Santiago-Cruz, S. D. Gennaro, O. Mitrofanov, S. Addamane, J. Reno, I. Brener, M. V. Chekhova, Resonant metasurfaces for generating complex quantum states. *Science* **377**, 991–995 (2022).

35. V. Boyer, A. M. Marino, R. C. Pooser, P. D. Lett, Entangled images from four-wave mixing. *Science* **321**, 544–547 (2008).

36. A. M. Lance, T. Symul, W. P. Bowen, B. C. Sanders, P. K. Lam, Tripartite quantum state sharing, *Phys. Rev. Lett.* **92**, 177903 (2004).

37. C. F. McCormick, V. Boyer, E. Arimondo, P. D. Lett, Strong relative intensity squeezing by four-wave mixing in rubidium vapor. *Opt. Lett.* **32**, 178–180 (2007).

38. M. Jasperse, L. D. Turner, R. E. Scholten, Relative intensity squeezing by four-wave mixing with loss: An analytic model and experimental diagnostic, *Opt. Express* **19**, 3765–3774 (2011).

39. L. M. Duan, G. Giedke, J. I. Cirac, P. Zoller, Inseparability criterion for continuous variable systems. *Phys. Rev. Lett.* **84**, 2722–2725 (2000).

40. R. Simon, Peres-Horodecki separability criterion for continuous variable systems. *Phys. Rev. Lett.* **84**, 2726–2729 (2000).

41. G. Adesso, A. Serafini, F. Illuminati, Quantification and scaling of multipartite entanglement in continuous variable systems, *Phys. Rev. Lett.* **93**, 220504 (2004).

42. F. A. S. Barbosa, A. S. Coelho, A. J. de Faria, K. N. Cassemiro, A. S. Villar, P. Nussenzveig, M. Martinelli, Robustness of bipartite Gaussian entangled beams propagating in lossy channels, *Nat. Photon.* **4**, 858–861 (2010).

43. X. Pan, S. Yu, Y. Zhou, K. Zhang, K. Zhang, S. Lv, S. Li, W. Wang, J. Jing, Orbital-angular-momentum multiplexed continuous-variable entanglement from four-wave mixing in hot atomic vapor, *Phys. Rev. Lett.* **123**, 070506 (2019).

44. Y. Chen, Q. Zhu, X. Wang , Y. Lou, S. Liu, J. Jing, Deterministic all-optical quantum state sharing, *Adv. Photonics* **5**, 026006 (2023).

45. A. M. Marino, R. C. Pooser, V. Boyer, P. D. Lett, Tunable delay of Einstein-Podolsky-Rosen entanglement, *Nature* **457**, 859–862 (2009).

46. B. Schumacher, Quantum coding. *Phys. Rev. A* **51**, 2738–2747 (1995).

47. A. M. Lance, T. Symul, W. P. Bowen, B. C. Sanders, T. Tyc, T. C. Ralph, P. K. Lam, Continuous-variable quantum-state sharing via quantum disentanglement. *Phys. Rev. A* **71**, 033814 (2005).

48. V. Boyer, A. M. Marino, P. D. Lett, Generation of spatially broadband twin beams for quantum imaging, *Phys. Rev. Lett.* **100**, 143601 (2008).

49. J.-S. Park, S. W. D. Lim, A. Amirzhan, H. Kang, K. Karrfalt, D. Kim, J. Leger, A. Urbas, M. Ossiander, Z. Li, F. Capasso, All-glass 100 mm diameter visible metalens for imaging the cosmos, *ACS Nano* **18**, 3187–3198 (2024).

50. Z. Li, R. Pestourie, J.-S. Park, Y.-W. Huang, S. G. Johnson, F. Capasso, Inverse design enables large-scale high-performance meta-optics reshaping virtual reality, *Nat. Commun.* **13**, 2409 (2022).

51. T. Tyc, D. J. Rowe, B. C. Sanders, Efficient sharing of a continuous-variable quantum secret. *J. Phys. A: Math. Gen.* **36**, 7625–7637 (2003).

52. A. Arbabi, E. Arbabi, Y. Horie, S. M. Kamali, A. Faraon, Planar metasurface retroreflector, *Nat. Photon.* **11**, 415–420 (2017).

53. S. T. Ha, Q. Li, J. K. W. Yang, H. V. Demir, M. L. Brongersma, A. I. Kuznetsov, Optoelectronic metadevices. *Science* **386**, eadm7442 (2024).

54. S. M. Kamali, E. Arbabi, A. Arbabi, A. Faraon, A review of dielectric optical metasurfaces for wavefront control. *Nanophotonics* **7**, 1041–1068 (2018).

55. S. Wengerowsky, S. K. Joshi, F. Steinlechner, H. Hübel, R. Ursin, An entanglement-based wavelength-multiplexed quantum communication network. *Nature* **564**, 225–228 (2018).

56. S. Liu, Y. Lv, X. Wang, J. Wang, Y. Lou, J. Jing, Deterministic all-optical quantum teleportation of four degrees of freedom. *Phys. Rev. Lett.* **132**, 100801 (2024).

57. S.-W. Moon, J. Kim, C. Park, W. Kim, Y. Yang, J. Kim, S. Lee, M. Choi, H. Sung, J. Park, H. Song, H. Lee, J. Rho, Wafer-scale manufacturing of near-infrared metalenses, *Laser Photonics Rev.* **18**, 2300929 (2024).

58. J. Kim, J. Seong, W. Kim, G.-Y. Lee, S. Kim, H. Kim, S.-W. Moon, D. K. Oh, Y. Yang, J. Park, J. Jang, Y. Kim, M. Jeong, C. Park, H. Choi, G. Jeon, K. Lee, D. H. Yoon, N. Park, B. Lee, H. Lee, J. Rho, Scalable manufacturing of high-index atomic layer–polymer hybrid metasurfaces for metaphotonics in the visible, *Nat. Mater.* **22**, 474–481 (2023).

59. N. Huo, Y. Liu, J. Li, L. Cui, X. Chen, R. Palivela, T. Xie, X. Li, Z. Y. Ou, Direct temporal mode measurement for the characterization of temporally multiplexed high dimensional quantum entanglement in continuous variables, *Phys. Rev. Lett.* **124**, 213603 (2020).

60. S. Shi, L. Tian, Y. Wang, Y. Zheng, C. Xie, K. Peng, Demonstration of channel multiplexing quantum communication exploiting entangled sideband modes, *Phys. Rev. Lett.* **125**, 070502 (2020).

61. Z. Yang, M. Jahanbozorgi, D. Jeong, S. Sun, O. Pfister, H. Lee, and X. Yi, A squeezed quantum microcomb on a chip, *Nat. Commun.* **12**, 4781 (2021).
